# Extracellular vesicles as biomimetic platforms for photo-assisted cancer therapy: evolution from synthetic nanocarriers to engineered therapeutic systems

**DOI:** 10.3389/fonc.2026.1955178

**Published:** 2026-09-22

**Authors:** Lucía Beaugé, Luis Exequiel Ibarra

**Affiliations:** 1 Departamento de Biología Molecular, Facultad de Ciencias Exactas, Fisicoquímicas y Naturales, Universidad Nacional de Río Cuarto (UNRC), Río Cuarto, Argentina; 2 Instituto de Investigaciones en Tecnologías Energéticas y Materiales Avanzados (IITEMA), UNRC y Consejo Nacional de Investigaciones Científicas y Técnicas (CONICET), Río Cuarto, Argentina; 3 Instituto de Biotecnología Ambiental y Salud (INBIAS), UNRC y CONICET, Río Cuarto, Argentina

**Keywords:** biomimetic drug delivery, extracellular vesicles, photo-assisted therapy, photodynamic therapy, theranostics, tumor microenvironment

## Abstract

Photo-assisted therapies have emerged as promising strategies for cancer treatment by combining localized tumor ablation with the induction of systemic antitumor immune responses. Despite encouraging preclinical and clinical advances, their therapeutic efficacy remains limited by the unfavorable pharmacokinetics, poor tumor selectivity, restricted tissue penetration, and off-target toxicity of conventional photosensitizer formulations. Although synthetic nanocarriers have substantially improved photosensitizer delivery, their limited biological functionality has driven the development of biomimetic drug delivery systems capable of actively interacting with the tumor microenvironment. Among these, extracellular vesicles have rapidly emerged as highly attractive therapeutic platforms owing to their intrinsic biocompatibility, prolonged circulation, natural tissue tropism, ability to cross biological barriers, and unique role in intercellular communication. In this review, we examine the evolution of extracellular vesicle-based photo-assisted therapeutic platforms from passive drug carriers to engineered biomimetic systems integrating multiple biological and therapeutic functions. We first discuss the transition from synthetic nanocarriers to extracellular vesicles and summarize the biological properties that distinguish these natural nanocarriers from conventional delivery systems. We then analyze current engineering strategies for optimizing therapeutic cargo loading and programming the extracellular vesicle biological interface through chemical, genetic, and biomimetic approaches that enhance tumor targeting, intracellular trafficking, and communication with the tumor microenvironment. Building upon these advances, we propose a functional framework describing the progressive evolution of extracellular vesicle-based photo-assisted platforms from targeted delivery systems to multifunctional nanotherapeutics integrating immune modulation, ferroptosis induction, metabolic reprogramming, hypoxia alleviation, image-guided therapy, and theranostic and image-guided capabilities. Finally, we discuss the major biological, technological, manufacturing, and regulatory barriers that currently limit clinical translation and highlight emerging opportunities for developing engineered extracellular vesicles capable of sensing, communicating with, and dynamically remodeling the tumor ecosystem. Together, this review provides a critical overview of current developments in EV-based photo-assisted cancer therapy, distinguishing experimentally demonstrated engineering strategies from emerging concepts whose translational feasibility remains to be established. Despite these advances, EV-based photo-assisted therapeutic platforms remain at the preclinical stage, with no clinical trials yet evaluating their therapeutic use in humans.

## 1 Introduction

Photo-assisted therapies have emerged as powerful therapeutic modalities for cancer treatment by enabling localized cytotoxicity with minimal damage to surrounding healthy tissues. Among them, photodynamic therapy (PDT) has become the most extensively investigated and clinically translated approach, relying on the activation of a photosensitizer (PS) by light of an appropriate wavelength to generate reactive oxygen species (ROS), predominantly singlet oxygen (^1^O_2_), that induce oxidative damage to tumor cells, vascular disruption, and immunogenic cell death (ICD) (1–3). During the last decade, the field has rapidly evolved beyond conventional PDT to encompass photothermal therapy (PTT) (4), sonodynamic therapy (SDT) (5, 6), photoimmunotherapy (7) and multimodal therapeutic strategies integrating chemotherapy, ferroptosis induction, gene therapy and immune modulation, considerably expanding the therapeutic potential of light-triggered cancer treatment (8, 9).

Despite these advances, the clinical efficacy of photo-assisted therapies remains largely determined by the efficiency with which therapeutic agents reach and accumulate within tumors. In this sense, most clinically approved and experimental PSs suffer from poor aqueous solubility, aggregation under physiological conditions, unfavorable pharmacokinetics, limited tumor selectivity, prolonged skin photosensitivity and heterogeneous intratumoral distribution, all of which compromise therapeutic efficacy. Consequently, the development of efficient drug delivery systems has become one of the major challenges in photo-assisted oncology (10–12).

Over the past two decades, nanotechnology has revolutionized drug delivery through the development of liposomes, polymeric nanoparticles, dendrimers, mesoporous silica nanoparticles, inorganic nanomaterials and numerous hybrid nanocarriers. These synthetic systems have significantly improved the solubility, stability and tumor accumulation of PSs while enabling multifunctional therapeutic platforms (12–14). Nevertheless, their clinical translation continues to be hindered by rapid clearance by the mononuclear phagocyte system, protein corona formation, limited penetration into dense tumor tissues, poor transport across biological barriers such as the blood-brain barrier, manufacturing complexity and insufficient biological responsiveness (15–17).

The growing recognition that naturally occurring biological nanostructures have evolved to overcome many of these limitations has shifted considerable attention toward biomimetic drug delivery systems (18, 19). Among them, extracellular vesicles (EVs) have emerged as one of the most promising endogenous nanoplatforms because of their intrinsic biocompatibility, low immunogenicity, prolonged circulation time, natural cargo protection, and highly efficient intercellular communication (20). Unlike synthetic nanoparticles, EVs inherit membrane lipids, proteins, glycans and signaling molecules from their parental cells, conferring unique biological properties that facilitate immune evasion, cellular uptake, tissue tropism and transport across biological barriers (21). These characteristics have positioned EVs as attractive carriers for a broad range of therapeutic compounds, including small molecules, proteins, nucleic acids, catalytic nanoparticles and, more recently, PSs for photo-assisted cancer therapy (10, 15, 22).

The use of EVs in photo-assisted therapy was initially driven by their ability to improve the loading and delivery of hydrophobic PSs. Fuhrmann et al. demonstrated that active loading into EVs significantly enhanced cellular uptake and photodynamic activity of porphyrins compared with free or liposome-encapsulated formulations (23). Subsequent studies showed that EV-based delivery could improve the stability, penetration, and phototoxic efficacy of mTHPC in three-dimensional tumor models, outperforming both free and liposomal formulations (10). These early studies established the conceptual basis for considering EVs not merely as natural vehicles, but as functional carriers for PS delivery.

More recent work has substantially expanded this concept. EVs have been engineered through passive and active loading, sonication, electroporation, endogenous biosynthesis, membrane fusion, bioorthogonal chemistry, peptide modification, hybridization with liposomes or nanoparticles, and genetic or cellular engineering (15, 24–27).

More recent studies have expanded the role of EVs beyond PS transport by incorporating functions such as tumor targeting, microenvironment modulation, immune regulation, hypoxia alleviation, and the induction of regulated cell death pathways (28–32). These advances support an emerging conceptual transition in which EVs are increasingly investigated not only as delivery vehicles, but also as biologically active components of photo-assisted therapeutic systems. We use this transition as the basis for a functional framework that organizes EV-based platforms according to increasing levels of engineering and therapeutic integration (**Figure 1**).

**Figure 1:**
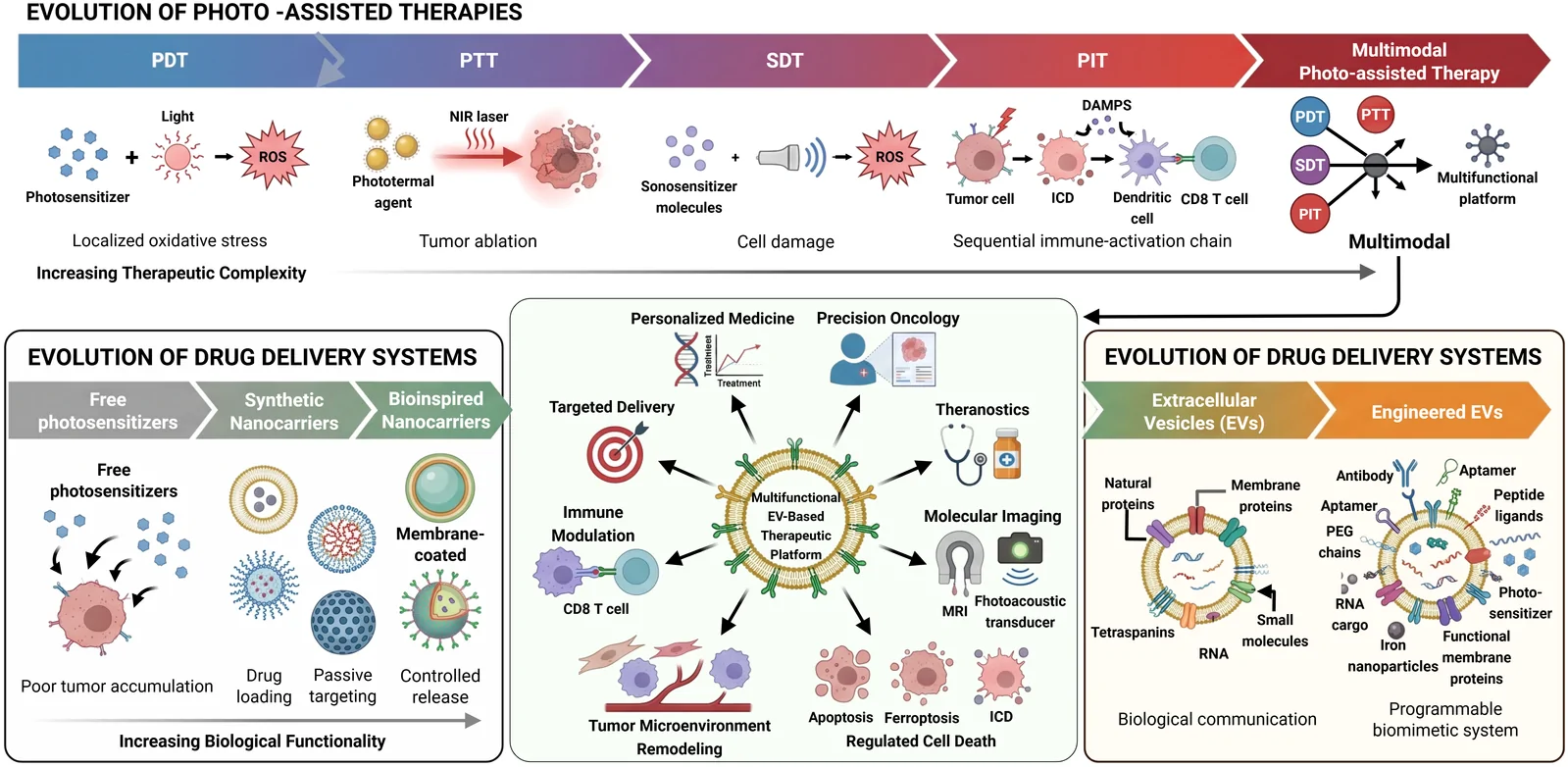
Convergence of photo-assisted cancer therapies and biomimetic drug delivery systems toward extracellular vesicle-based therapeutic platforms. The upper axis depicts the diversification of photo-assisted cancer therapies from photodynamic therapy (PDT) toward photothermal therapy (PTT), sonodynamic therapy (SDT), photoimmunotherapy (PIT), and multimodal approaches. The lower axis illustrates the parallel evolution of delivery systems from free PSs and synthetic nanocarriers toward bioinspired and extracellular vesicle (EV)-based platforms. Their convergence supports the integration of targeted delivery, imaging, tumor microenvironment (TME) modulation, immune regulation, and regulated cell death within multifunctional therapeutic systems. Created in BioRender.

In this review, we critically analyze the evolution of EVs as delivery platforms for photo-assisted cancer therapy, with particular emphasis on PDT, using a technology-oriented framework that follows the progression from conventional synthetic nanocarriers to engineered biomimetic EV-based systems. We discuss advances in EV engineering, PS loading strategies, multimodal therapeutic applications, interactions with the TME, translational challenges, and emerging opportunities that may define the next generation of intelligent nanotheranostic platforms for precision oncology.

## 2 From synthetic nanocarriers to biomimetic drug delivery

The integration of nanotechnology into photo-assisted cancer therapy has substantially improved the formulation and delivery of PSs. Many PSs exhibit poor aqueous solubility, molecular aggregation, limited stability, unfavorable pharmacokinetics, and insufficient tumor selectivity, prompting the development of nanoscale delivery systems capable of improving their pharmaceutical properties. Liposomes, polymeric nanoparticles, micelles, dendrimers, mesoporous silica nanoparticles, metallic nanostructures, and metal-organic frameworks have consequently been investigated as carriers for PS delivery, enabling improved solubility, protection from degradation, controlled release, and combination with imaging or complementary therapeutic modalities (13, 33–36). Encapsulation within nanoscale delivery systems significantly improved drug stability, pharmacokinetics, and tumor accumulation, establishing nanomedicine as a fundamental component of modern PDT (37).

Synthetic nanocarriers retain several important advantages that should not be underestimated. Their composition, size, surface charge, drug-to-carrier ratio, and release kinetics can generally be controlled more precisely than those of biologically derived vesicles (38). Established formulation technologies also permit relatively reproducible production, incorporation of high payloads, and modular surface functionalization. These characteristics make synthetic systems particularly attractive when pharmaceutical reproducibility, physicochemical control, and scalable manufacturing are dominant design requirements. Stimuli-responsive materials have further expanded their capabilities by enabling release in response to pH, redox conditions, enzymes, hypoxia, magnetic fields, ultrasound, or light, thereby supporting increasingly sophisticated combinations of photodynamic, photothermal, sonodynamic, chemotherapeutic, and imaging modalities (3, 39–42).

Nevertheless, optimizing physicochemical performance does not necessarily ensure efficient biological delivery. Following systemic administration, synthetic nanoparticles encounter protein adsorption and corona formation, recognition by the mononuclear phagocyte system, heterogeneous tumor vasculature, elevated interstitial pressure, dense extracellular matrix, and limited transport across specialized barriers such as the blood–brain barrier. These factors can substantially alter nanoparticle identity and biodistribution and may reduce the fraction of the administered dose that ultimately reaches therapeutically relevant tumor compartments (43–46). Extensive surface engineering can partially mitigate these limitations, but increased structural complexity may simultaneously complicate manufacturing, characterization, reproducibility, and regulatory assessment.

These challenges have stimulated interest in biomimetic delivery systems that incorporate biological components into otherwise synthetic platforms. Cell membrane-coated nanoparticles, bacterial membrane vesicles, virus-inspired particles, hybrid lipid–biological systems, and EVs represent different points along this spectrum. EVs are particularly attractive because their membranes contain proteins, lipids, glycans, adhesion molecules, and signaling components inherited from their parental cells, which may contribute to cellular uptake, tissue interactions, cargo protection, and intercellular communication. Such functions are difficult to reproduce simultaneously using synthetic materials alone and may be advantageous when biological recognition, barrier interaction, or communication with the tumor microenvironment (TME) is central to therapeutic efficacy.

However, the biological origin of EVs also introduces limitations that contrast directly with the engineering advantages of synthetic carriers. EV preparations are intrinsically heterogeneous, and their molecular composition can vary according to donor-cell type, culture conditions, physiological state, isolation procedure, and production batch. Drug loading is often less predictable than in synthetic nanocarriers, while purification, characterization, storage, potency assessment, and large-scale production remain technically demanding. Furthermore, the biological activity inherited from parental cells is not invariably beneficial: tumor-derived or immune-cell-derived EVs may carry signaling molecules capable of producing unintended biological effects, and extensive engineering may alter biodistribution or immunological behavior. Thus, biological complexity constitutes both a major strength and a major translational liability of EV-based systems.

Accordingly, EVs should not be considered universally superior replacements for synthetic nanocarriers. Rather, the two platform classes offer complementary advantages and limitations (**Figure 2**). Synthetic nanocarriers provide greater compositional control, loading flexibility, reproducibility, and manufacturing maturity, whereas EVs provide biological interactions and source-dependent functionalities that are difficult to engineer synthetically. The optimal platform therefore depends on the therapeutic cargo, administration route, tumor biology, desired targeting mechanism, required degree of biological interaction, and feasibility of manufacturing and quality control. Increasingly, hybrid biomimetic systems seek to integrate these complementary properties by combining the engineering precision of synthetic materials with selected biological functions of cell-derived vesicles.

**Figure 2:**
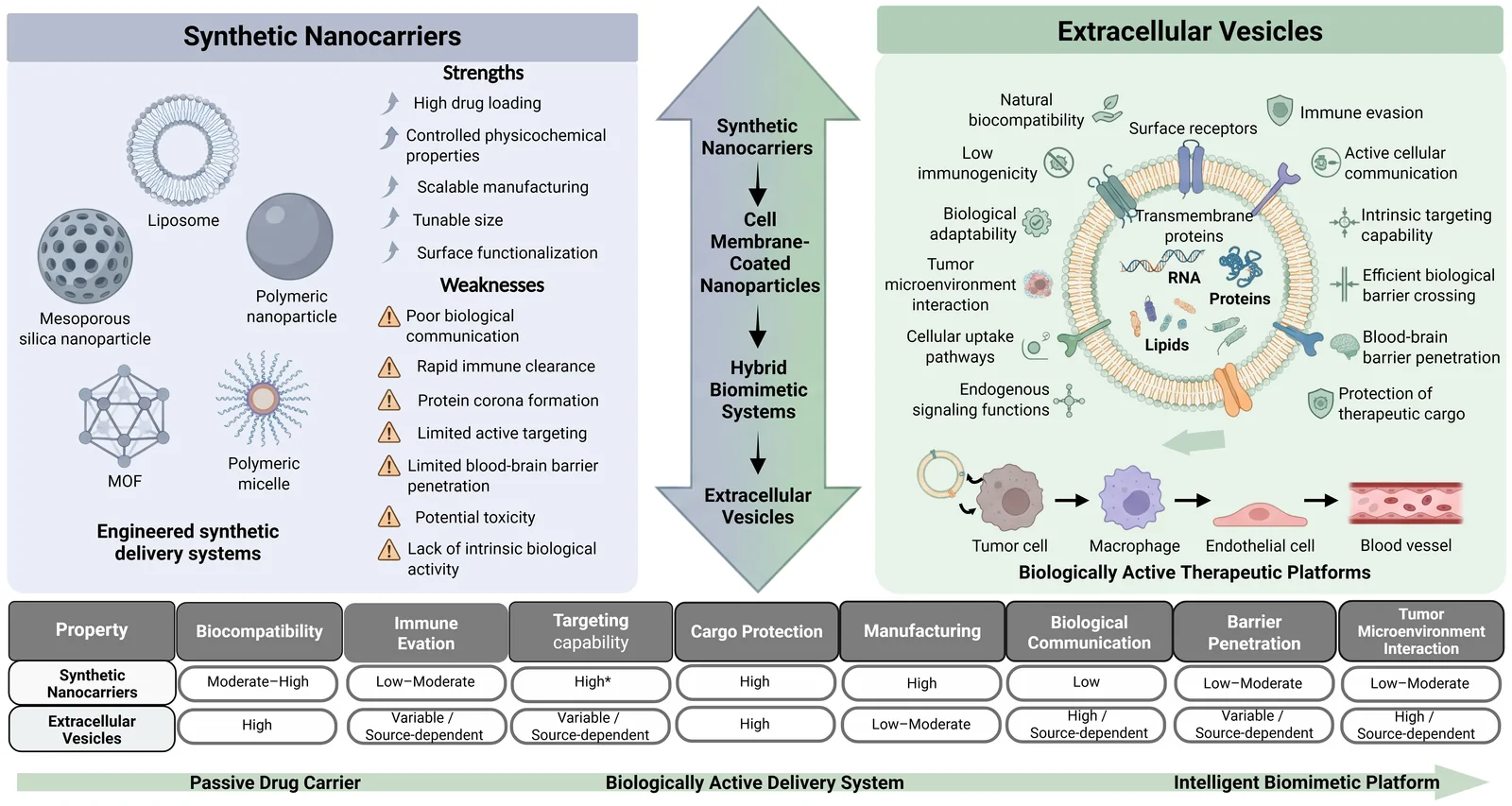
Comparative strengths and limitations of synthetic nanocarriers and extracellular vesicles for photo-assisted cancer therapy. Synthetic nanocarriers provide high physicochemical control, flexible drug loading, reproducible formulation, and comparatively mature manufacturing, whereas EVs provide endogenous biological interfaces and source-dependent cellular interactions. EVs nevertheless present challenges related to heterogeneity, loading control, purification, batch reproducibility, and scalable production. Hybrid biomimetic systems seek to combine complementary properties of both platform classes. Created in BioRender.

Among these platforms, EVs have emerged as perhaps the most versatile and biologically sophisticated delivery system currently available. Initially investigated as naturally occurring nanocarriers capable of improving the transport of hydrophobic PSs, EVs rapidly demonstrated advantages extending far beyond passive drug delivery. Their endogenous membrane composition, inherited surface proteins, glycans, lipids, and intrinsic cell-derived signaling molecules confer unique biological properties, including efficient cellular internalization, prolonged circulation, immune evasion, tissue tropism, and intercellular communication, features that remain difficult to reproduce using entirely synthetic materials (10, 23, 47).

More importantly, advances in bioengineering have progressively transformed EVs into multifunctional therapeutic platforms through endogenous cargo loading, membrane engineering, peptide functionalization, bioorthogonal chemistry, hybridization with synthetic nanoparticles, genetic modification, and stimuli-responsive designs (24, 25, 27).

Consequently, the role of the delivery system itself has fundamentally changed. Modern EV-based platforms are no longer designed solely to transport therapeutic molecules but to actively participate in treatment by remodeling the TME (48), promoting ICD (49), modulating macrophage polarization (50), reprograming TME (51), enhancing tumor penetration (52), inducing ferroptosis (53), and integrating multimodal imaging with precision therapy (54).

EVs should therefore be viewed as complementary rather than universally superior alternatives to synthetic nanocarriers. Synthetic systems retain important advantages in terms of compositional control, physicochemical tunability, reproducibility, drug-loading capacity, and scalable manufacturing, whereas EVs offer biological properties that are difficult to reproduce synthetically, including endogenous molecular recognition, intercellular communication, and source-dependent tissue interactions. The relative value of each platform consequently depends on the therapeutic objective, cargo characteristics, route of administration, and required level of biological functionality. Hybrid biomimetic systems increasingly seek to combine these complementary strengths while minimizing their respective limitations.

These trade-offs emphasize that the therapeutic value of EVs cannot be understood solely from their physicochemical characteristics; it is closely linked to the biological properties inherited from their cells of origin. The following section therefore examines these properties and their implications for photo-assisted cancer therapy.

## 3 Extracellular vesicles: the emergence of biologically active drug delivery systems

EVs have emerged as one of the most promising biomimetic nanoplatforms for drug delivery owing to a unique combination of biological and physicochemical properties that distinguish them from conventional synthetic nanocarriers. Originally recognized as mediators of intercellular communication, EVs are now understood to participate in virtually every aspect of tissue homeostasis and disease progression by transferring proteins, lipids, metabolites, messenger RNAs, microRNAs, DNA fragments, and other bioactive molecules between donor and recipient cells. This intrinsic biological function provides EVs with an exceptional ability to interact dynamically with living systems, making them attractive candidates for therapeutic delivery in oncology (15, 55).

Unlike artificial nanoparticles, EVs are naturally produced through highly regulated intracellular pathways. Small EVs, commonly referred to as exosomes, originate from the endosomal system through the formation of multivesicular bodies, whereas larger microvesicles are generated by direct outward budding of the plasma membrane (56). Although these biogenesis pathways differ mechanistically, both types of vesicles retain molecular components inherited from their parental cells, including membrane proteins, phospholipids, cholesterol, sphingolipids, glycoconjugates, adhesion molecules, tetraspanins, heat shock proteins, and numerous signaling molecules. Thus, EVs are not biologically inert carriers; instead, they are dynamic entities that retain a portion of the biological identity of their originating cells.

This cellular inheritance represents one of the major conceptual differences between EVs and synthetic nanocarriers. While conventional nanoparticles require extensive surface engineering to achieve efficient cellular recognition or tissue targeting, EVs naturally display membrane-associated molecules capable of interacting with specific receptors on recipient cells. Tetraspanins, integrins, proteoglycans, phosphatidylserine, lectin-binding glycans, and adhesion proteins contribute to highly efficient cellular internalization through multiple mechanisms, including clathrin-dependent endocytosis, caveolae-mediated uptake, macropinocytosis, phagocytosis, and, in certain circumstances, direct membrane fusion (57, 58). The coexistence of multiple uptake pathways increases delivery efficiency while reducing dependence on a single internalization mechanism, thereby improving therapeutic robustness across different tumor types.

Another defining characteristic of EVs is their remarkable capacity to preserve and protect complex molecular cargoes during systemic circulation. Their phospholipid bilayer efficiently shields proteins, nucleic acids, enzymes, PSs, and nanoparticles from enzymatic degradation and premature clearance (59–61). Unlike many synthetic formulations, which frequently require chemical stabilizers or protective polymers, EV membranes provide an endogenous protective environment that contributes to prolonged cargo stability while maintaining biological activity after delivery to recipient cells.

Perhaps the most distinctive feature of EVs is their ability to actively communicate with the biological microenvironment. EVs also participate in the continuous exchange of molecular information among tumor, stromal, endothelial, and immune cells, as well as distant organs. This communication regulates angiogenesis, immune responses, extracellular matrix remodeling, metabolic adaptation, and metastatic niche formation (62, 63). Although these processes frequently contribute to tumor progression, they also offer unique therapeutic opportunities by enabling engineered EVs to exploit endogenous communication pathways for selective drug delivery and immune modulation (64, 65).

These biological properties become particularly advantageous in photo-assisted cancer therapy. Effective photodynamic treatment requires not only sufficient accumulation of PSs within tumors but also efficient intracellular localization, homogeneous penetration throughout the tumor mass, and prolonged retention before light activation. By naturally interacting with recipient cells and protecting therapeutic cargoes during circulation, EVs may significantly improve intracellular PS delivery while reducing premature leakage and nonspecific distribution.

A further benefit of EVs is their ability to cross biological barriers that pose difficulties for traditional nanocarriers. Numerous studies have shown that EVs may cross endothelial barriers, infiltrate thick extracellular matrices, and, contingent upon their biological origin, display improved transit across the blood-brain barrier. The aforementioned qualities have sparked significant interest in using engineered EVs for the treatment of brain cancers, especially glioblastoma, where insufficient drug delivery is a primary factor contributing to therapeutic failure (31, 32).

Importantly, the biological performance of EVs is not universal but strongly depends on their cellular origin. Vesicles derived from mesenchymal stromal cells, macrophages, dendritic cells, tumor cells, natural killer cells, milk, plants, and other biological sources differ substantially in membrane composition, biodistribution, immunological properties, and therapeutic potential. This biological diversity can be therapeutically exploited by selecting parental cells with desirable characteristics or by engineering vesicles to acquire specific functionalities.

Nevertheless, native EVs also present intrinsic limitations that should not be overlooked. Heterogeneity in vesicle composition, variability among donor cells, limited drug loading capacity, low production yields, incomplete understanding of biodistribution, and the lack of standardized isolation and characterization protocols continue to hinder clinical translation. These challenges have stimulated the rapid development of EV engineering strategies aimed at enhancing cargo loading efficiency, improving targeting specificity, increasing therapeutic potency, and enabling scalable manufacturing. (66–68),

EV source represents another important design variable rather than a neutral manufacturing choice. Tumor-derived EVs may provide homotypic recognition and efficient interaction with malignant cells but can retain tumor-associated molecules with potentially undesirable biological effects. Immune-cell-derived EVs can contribute intrinsic immunomodulatory functions and may be particularly attractive for photoimmunotherapeutic strategies, although their biological activity may vary with the activation state of the parental cells. Mesenchymal stromal cell-derived EVs offer comparatively established production workflows and broad therapeutic versatility, but their tumor-targeting properties are context dependent and their immunomodulatory effects may not always be desirable in cancer. Plant- or food-derived vesicles may provide advantages in accessibility and scalability, while their biodistribution, molecular composition, and regulatory classification remain less established for systemic cancer therapy. EV source selection should therefore be guided by the biological function required from the carrier rather than by availability alone.

These limitations have stimulated the development of engineering strategies aimed at improving cargo loading, targeting specificity, structural stability, therapeutic potency, and manufacturing reproducibility. The following section therefore examines EV engineering according to the specific level at which intervention occurs—therapeutic cargo, membrane architecture, biological interface, or integrated therapeutic function—while considering the advantages and trade-offs associated with each strategy.

## 4 Engineering extracellular vesicles for photo-assisted therapy

Engineering strategies can modify EV performance at several interconnected levels, including therapeutic cargo, membrane architecture, biological interactions, and integrated therapeutic function. These levels provide a useful framework for examining how individual modifications influence loading efficiency, biodistribution, cellular recognition, intracellular trafficking, and therapeutic outcome (**Figure 3**). Importantly, increasing engineering complexity does not necessarily translate into superior therapeutic performance, because each additional modification may introduce trade-offs related to vesicle integrity, reproducibility, immunogenicity, manufacturing complexity, and regulatory feasibility.

**Figure 3:**
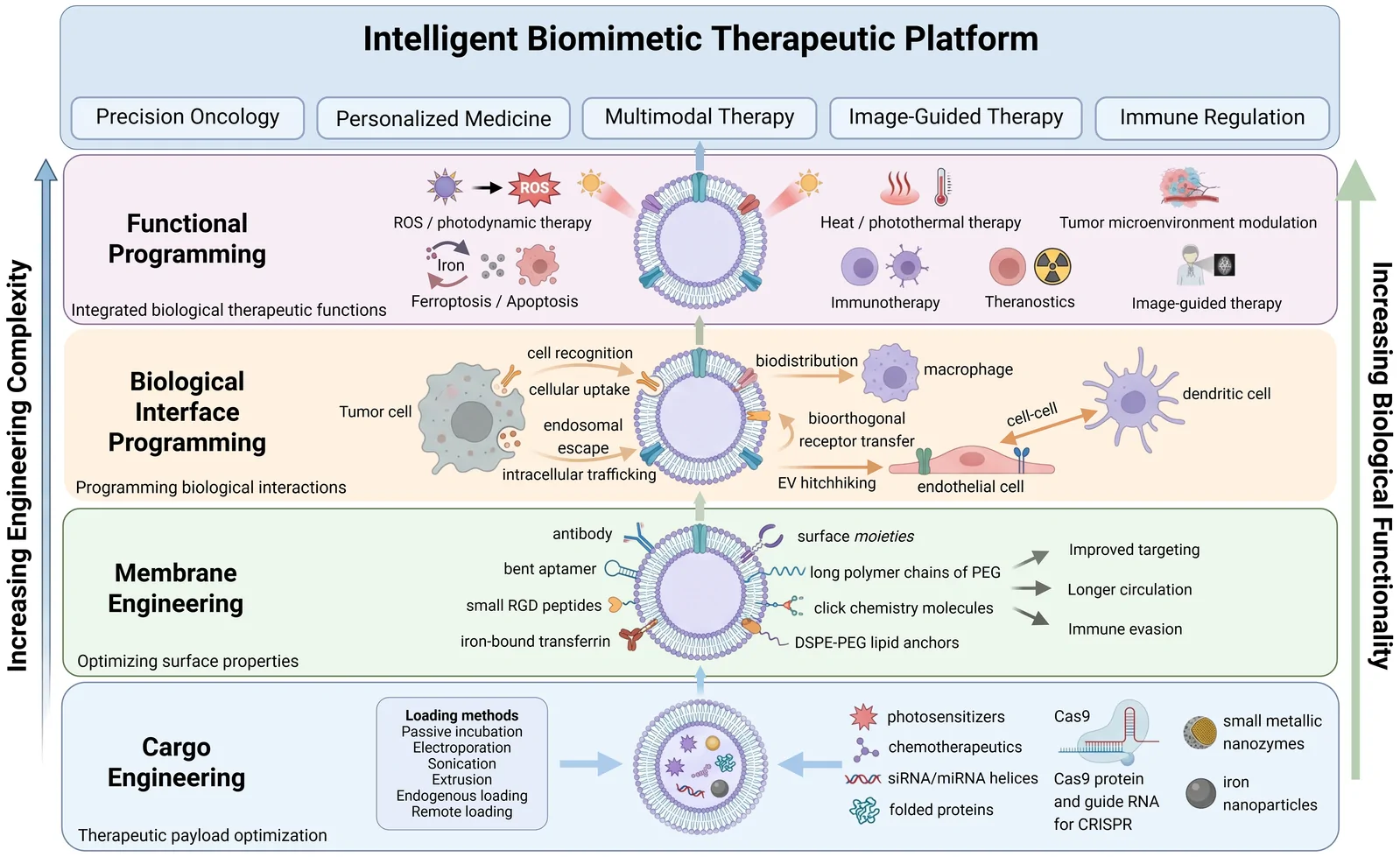
Hierarchical engineering of extracellular vesicles for photo-assisted cancer therapy. EV engineering is organized into four interconnected levels: cargo engineering, membrane engineering, biological interface programming, and functional programming. These levels describe modifications affecting therapeutic payload, surface properties, biological interactions, and integrated therapeutic functions, respectively. The levels are interconnected but not obligatory, and the optimal degree of engineering depends on the intended therapeutic application. Created in BioRender.

### 4.1 Engineering the cargo: optimizing therapeutic payloads

The earliest engineering strategies primarily focused on improving the incorporation of therapeutic agents into EVs. PSs are often highly hydrophobic molecules with limited loading efficiency under physiological conditions. Consequently, considerable efforts have been directed toward maximizing cargo encapsulation while preserving EV integrity and biological activity.

Passive incubation represents the simplest and most widely used loading strategy. Hydrophobic PSs spontaneously partition into the phospholipid bilayer during co-incubation, allowing relatively straightforward preparation without substantially affecting membrane integrity. This approach proved sufficient for the incorporation of several porphyrin derivatives, including mTHPC and zinc phthalocyanines, and established the first proof-of-concept studies demonstrating superior photodynamic efficacy of EV-loaded PSs compared with free drugs or liposomal formulations (10, 23).

However, passive loading frequently results in variable encapsulation efficiencies, particularly for hydrophilic compounds, nucleic acids, or large biomolecules. To overcome these limitations, active loading approaches—including electroporation (69), sonication (70), extrusion, saponin permeabilization (71), freeze-thaw cycles, and hypotonic dialysis (23) —have been developed to transiently increase membrane permeability and facilitate cargo incorporation (15, 70).

More recently, endogenous loading strategies have emerged as particularly attractive alternatives. Endogenous loading follows a different strategy in which parental cells are exposed to PSs, prodrugs, nanoparticles, nucleic acids, or therapeutic drugs before EV secretion, allowing physiological intracellular sorting mechanisms to incorporate therapeutic cargo into newly generated vesicles (25, 72). Cell-mediated encapsulation often preserves membrane integrity more effectively than post-isolation loading while enabling simultaneous incorporation of multiple therapeutic components.

The complexity of therapeutic cargo has also evolved considerably. Whereas early studies focused exclusively on delivering PSs, engineered EVs now simultaneously transport chemotherapeutic drugs, immunomodulators, ferroptosis inducers, oxygen-generating nanoparticles, catalytic nanomaterials, proteins, CRISPR components, messenger RNA, siRNA, microRNAs, and imaging probes (26, 31, 32, 73).

An important conceptual advance in EV cargo engineering has been the adaptation of active remote-loading strategies, originally developed for clinically approved liposomal formulations. Inspired by the ammonium sulfate transmembrane gradient used to actively encapsulate doxorubicin into liposomes, Chen et al. developed the Sonication and Extrusion-assisted Active Loading (SEAL) strategy, which generates a stable transmembrane ion gradient within extracellular vesicles, enabling highly efficient drug accumulation while preserving vesicle integrity (66). Unlike conventional passive loading approaches, SEAL exploits physicochemical driving forces rather than transient membrane permeabilization, resulting in substantially higher loading efficiency, improved drug retention, and enhanced formulation stability. Although this methodology was validated using doxorubicin, its underlying mechanism is independent of the therapeutic payload and therefore represents an attractive platform for encapsulating hydrophobic PSs, porphyrins, phthalocyanines, chlorins, and even multifunctional therapeutic combinations involving PSs, ferroptosis inducers, or nucleic acids. The application of remote-loading strategies to EV-mediated photo-assisted therapy remains largely unexplored and may represent one of the most promising avenues for improving cargo loading efficiency while preserving the intrinsic biological properties of EVs.

The choice of loading strategy therefore depends on the physicochemical properties of the therapeutic cargo and on the degree of vesicle manipulation that can be tolerated. Passive incubation is experimentally simple and generally preserves EV integrity, but it is most suitable for hydrophobic molecules with favorable membrane partitioning and often provides limited loading efficiency for hydrophilic or macromolecular cargos. Active post-isolation approaches, including electroporation, sonication, and extrusion, can increase cargo incorporation and broaden the range of loadable molecules, although membrane disruption, cargo aggregation, and batch-to-batch variability may compromise EV quality. Endogenous loading through donor-cell exposure can better preserve membrane architecture and exploit physiological cargo-sorting mechanisms, but loading efficiency is less directly controlled and depends on cellular uptake, intracellular trafficking, and potential cargo toxicity to the parental cell. Consequently, no loading method is universally optimal, and strategy selection should balance loading efficiency, cargo compatibility, preservation of EV biological properties, and manufacturing reproducibility.

### 4.2 Engineering the EV biological interface: programming biological recognition and communication

Among the different levels of EV engineering, membrane modification represents one of the most powerful strategies for controlling the biological fate of extracellular vesicles following systemic administration. Whereas cargo engineering determines what therapeutic payload is delivered, membrane engineering dictates where, to which cells, and under which biological conditions this payload is delivered. Consequently, the EV membrane should no longer be regarded as a passive lipid bilayer enclosing therapeutic cargo but rather as a programmable biological interface capable of orchestrating multiple stages of the therapeutic process, including biodistribution, cellular recognition, intercellular communication, and responsiveness to the TME.

Native EVs inherently display a wide repertoire of membrane-associated biomolecules—including integrins, tetraspanins (CD9, CD63 and CD81), glycans, phosphatidylserine, adhesion molecules and membrane receptors—that facilitate selective interactions with recipient cells and contribute to their intrinsic tissue tropism. Nevertheless, these naturally evolved recognition mechanisms are not necessarily optimal for therapeutic applications, particularly when efficient and selective delivery to tumor tissues is required. Consequently, considerable efforts have been devoted to engineering the EV membrane in order to enhance targeting specificity while preserving its biological functionality. A wide variety of active targeting strategies have been developed through both genetic engineering of parental cells and post-isolation chemical modification. Unlike synthetic nanoparticles, whose surface ligands are generally introduced through purely chemical conjugation, the EV membrane offers the possibility of biologically oriented ligand presentation (74). Because targeting molecules can be genetically expressed by parental cells or inserted into the membrane while maintaining their native orientation, membrane engineering can preserve receptor accessibility and biological activity more efficiently than conventional nanoparticle functionalization. This unique feature has enabled the development of increasingly sophisticated strategies based on chemical, physical and genetic membrane engineering.

#### 4.2.1 Chemical and physical engineering of the EV membrane

Post-isolation functionalization remains one of the most versatile approaches for modifying EV membranes because it enables rapid incorporation of targeting ligands without manipulating parental cells. Among these strategies, lipid insertion using amphiphilic molecules such as DSPE-PEG conjugates has become particularly attractive. The hydrophobic phospholipid tail spontaneously inserts into the EV membrane while the PEG chain exposes functional groups or targeting ligands toward the extracellular environment. This strategy has been successfully employed to decorate EVs with biotin, folic acid, peptides and other targeting molecules directed against tumor-associated receptors while minimally affecting vesicle integrity (25, 75).

Another major advance has been the application of bioorthogonal click chemistry, which enables highly selective covalent conjugation reactions under physiological conditions with minimal perturbation of membrane architecture. Compared with conventional chemical modification, click reactions provide superior efficiency, excellent biocompatibility and precise spatial control over ligand conjugation. For example, cyclo(Arg-Gly-Asp-D-Tyr-Lys) [c(RGDyK)] peptides have been conjugated onto EV membranes to enhance targeting of α_vβ_3 integrins overexpressed in tumor vasculature and ischemic tissues, significantly improving tissue accumulation after systemic administration (76).

Importantly, membrane engineering is increasingly evolving from simple receptor recognition toward dynamic biological targeting. Recent strategies increasingly focus on receptor accessibility, ligand orientation, and multivalent interactions to enhance binding avidity under physiological conditions. This transition reflects an important conceptual shift in EV engineering, where biological recognition is no longer viewed solely as receptor-ligand binding but as a programmable process integrating molecular orientation, membrane fluidity and cellular communication.

Physical membrane engineering has also been employed to improve therapeutic performance of PDT. Membrane fusion, extrusion, freeze-thaw cycling and sonication not only facilitate cargo incorporation but may also alter membrane composition and mechanical properties, influencing cellular uptake and intracellular trafficking. Hybrid membranes generated by fusing EVs with synthetic liposomes further expand these possibilities by combining the biological communication capabilities of native EVs with the high loading capacity and engineering flexibility of synthetic nanocarriers (26).

#### 4.2.2 Genetic engineering and cellular programming

While post-isolation surface modification provides considerable flexibility, genetic engineering of parental cells offers an alternative strategy in which the biological identity of EVs is programmed during their biogenesis. In this approach, donor cells are genetically modified to express membrane-associated proteins, receptors, antibodies, or signaling molecules that are subsequently incorporated into EVs during biogenesis. This preserves the native membrane topology and orientation of engineered proteins, ensuring optimal receptor accessibility while minimizing structural perturbations frequently associated with post-isolation conjugation techniques.

One of the earliest applications of this strategy involved the generation of genetically engineered plasma membrane nanovesicles (GNVs). In these systems, donor cells such as HEK293T are transfected with plasmids encoding antibodies, nanobodies or affibodies fused to membrane proteins. Following vesicle production, the resulting nanovesicles display targeting ligands with their correct extracellular orientation, providing highly reproducible ligand presentation while taking advantage of the cellular biosynthetic machinery (74).

More recently, immune-cell engineering has significantly expanded the therapeutic potential of EVs (19). Exosomes derived from chimeric antigen receptor-engineered natural killer (CAR-NK) cells inherit both the intrinsic cytotoxic characteristics of NK cells and the antigen specificity conferred by CAR expression. Wang and colleagues demonstrated that HER2-targeted CAR-NK exosomes efficiently recognized HER2-positive brain metastatic cells while simultaneously delivering PSs and ferroptosis-inducing agents, resulting in highly selective multimodal photo-assisted therapy (27).

Genetic engineering has also been exploited to manipulate the biological fate of EVs after systemic administration. Overexpression of CD47, a transmembrane protein that interacts with signal regulatory protein-α (SIRPα) on macrophages, significantly reduces phagocytic clearance by the mononuclear phagocyte system. By prolonging blood circulation and increasing the probability of tumor accumulation, CD47-engineered EVs improve therapeutic delivery without directly modifying targeting specificity (73). This study demonstrated that erastin and Rose Bengal PS could be efficiently encapsulated into exosomes during sonication, and the drug-loaded exosomes (Er/RB@ExosCD47) significantly promoted ferroptosis in tumor cells under irradiation with a 532 nm laser. Furthermore, in comparison to exosomes lacking CD47 (Er/RB@ExosCtrl), Er/RB@ExosCD47 exhibited significantly reduced hepatic toxicity.

These investigations collectively illustrate that parental cell engineering enhances membrane modification beyond traditional surface functionalization. Donor-cell engineering can generate EVs displaying predefined membrane-associated proteins or recognition properties during vesicle biogenesis with predetermined recognition characteristics, immunological compatibility, and therapeutic efficacy.

Post-isolation and donor-cell-based membrane engineering therefore offer complementary advantages. Chemical conjugation, lipid insertion, and bioorthogonal modification provide relatively rapid and modular control over EV surface composition and may be preferable when different targeting ligands must be screened without generating a new producer-cell line. However, excessive surface modification can perturb membrane organization, alter ligand orientation, or introduce additional purification requirements. Genetic engineering of donor cells can provide more physiologically oriented and potentially stable display of membrane proteins during EV biogenesis, making it particularly attractive for receptors, antibodies, or complex membrane-associated proteins. Its disadvantages include longer development times, variability in expression and EV incorporation, potential modification of donor-cell biology, and additional manufacturing and regulatory complexity. The preferred strategy therefore depends on the molecular nature of the surface modification, the required stability and orientation of the ligand, and the intended level of manufacturing complexity.

#### 4.2.3 Programming intracellular fate

Successful tumor targeting alone does not guarantee therapeutic efficacy. Following cellular internalization, the intracellular destination of the delivered PS largely determines the biological outcome of photodynamic therapy because reactive oxygen species act over extremely short diffusion distances. Consequently, recent engineering strategies have begun to integrate membrane targeting with subcellular delivery mechanisms. A representative example is the development of dual-targeting chimeric peptides containing a membrane-anchoring alkyl chain, protoporphyrin IX (PpIX), and a nuclear localization signal (NLS), which enables sequential delivery from the plasma membrane to the nucleus following photoactivation (47). This study illustrates how membrane engineering can regulate not only which cells receive therapeutic cargo but also its intracellular destination, thereby maximizing photodynamic efficacy. Because ROS generated during PDT possess extremely short diffusion distances, precise subcellular localization of PSs can profoundly influence treatment outcome. Future engineering strategies will therefore likely emphasize the integration of membrane targeting with intracellular trafficking signals capable of directing therapeutic cargo toward organelles that maximize oxidative damage while minimizing off-target toxicity.

#### 4.2.4 Programming intercellular communication

Perhaps the most transformative recent advance in membrane engineering is the realization that EVs can be designed not only to recognize recipient cells but also to actively manipulate intercellular communication networks within tumors. This concept extends the function of EVs beyond one-time drug delivery toward dynamic propagation of therapeutic information throughout the tumor ecosystem.

A pioneering example was provided by Wang et al., who developed metabolically engineered EVs capable of transferring artificial azide-containing receptors to tumor cell membranes (24). These engineered vesicles extended conventional receptor-mediated targeting by installing new bioorthogonal docking sites on recipient cells, thereby enabling a second wave of selective nanoparticle targeting. This work introduced the concept of biological receptor propagation, in which EVs actively modify the molecular landscape of tumors to facilitate subsequent therapeutic interventions.

More recently, this concept has evolved further with the development of extracellular vesicle hitchhiking strategies. Instead of relying exclusively on direct nanoparticle penetration, therapeutic nanomaterials are designed to transiently associate with endogenous EVs naturally released by tumor cells. These endogenous vesicles subsequently transport therapeutic cargo deep into poorly vascularized and hypoxic tumor regions, markedly improving tissue penetration and amplifying antitumor efficacy (77). By exploiting the physiological communication network already established within tumors, EV hitchhiking transforms endogenous vesicles into secondary biological transporters rather than passive bystanders.

Together, these studies suggest that membrane engineering has evolved from optimizing cell targeting toward programming biological communication, where engineered EVs actively reshape information flow within the TME to amplify therapeutic dissemination.

#### 4.2.5 Programming the tumor microenvironment

The most recent stage in membrane engineering extends beyond improving drug delivery to actively modulating the biological behavior of the TME. Engineered EVs can also exploit their biological origin to influence stromal, vascular, and immune-cell populations that collectively determine therapeutic outcome.

One important example is provided by tumor-derived extracellular vesicles (TEXs). Because these vesicles retain membrane proteins, adhesion molecules and integrins inherited from their parental tumor cells, they exhibit pronounced homotypic tropism, preferentially interacting with cells originating from the same tumor. Although native TEXs are frequently associated with tumor progression and immune suppression, this intrinsic recognition capacity can be therapeutically exploited by loading TEXs with PSs or anticancer drugs, effectively transforming them into biological “Trojan horses” capable of selectively delivering therapeutic cargo back to the tumor (78, 79).

Conversely, EVs derived from immune cells can be engineered to reshape the immunological landscape of tumors. In particular, M1 macrophage-derived EVs inherit pro-inflammatory membrane components and cytokine-associated signaling molecules that promote macrophage repolarization, dendritic cell activation and cytotoxic T-cell recruitment. When combined with PDT, these vesicles not only improve tumor targeting but also amplify PDT-induced ICD, thereby converting local oxidative damage into systemic antitumor immunity (28, 80).

Finally, membrane engineering is increasingly incorporating stimuli-responsive biological interfaces capable of responding to the biochemical characteristics of the TME. ROS-sensitive thioketal (TK) linkers, for example, can be used to conceal targeting ligands beneath detachable PEG shells that are selectively removed in the oxidative environment generated during PDT (26). This strategy minimizes nonspecific interactions during systemic circulation while exposing targeting ligands only after accumulation within tumors, thereby increasing therapeutic specificity and reducing off-target toxicity.

These advancements collectively demonstrate that the biological interface of extracellular vesicles is no longer only aimed at enhancing biodistribution or cellular absorption. Contemporary membrane engineering increasingly seeks to program the tumor ecosystem by merging selective identification, immune regulation, microenvironment modification, and controlled therapeutic activation into a unified multifunctional biomimetic platform.

The remarkable diversity of engineering approaches developed for EVs reflects the rapid evolution of the field from simple drug encapsulation toward sophisticated biomimetic platform design. Depending on the therapeutic objective, engineering strategies can be directed at optimizing cargo loading, modifying membrane composition, programming biological interactions, or integrating multiple functionalities within a single vesicle. These approaches are complementary and can be combined to improve targeting specificity, therapeutic efficacy, immune modulation, and multimodal functionality. **Table 1** summarizes the principal engineering strategies currently employed for EV-based photo-assisted therapy, highlighting their underlying mechanisms, major advantages, current limitations, and representative applications.

**Table 1:** Engineering strategies for extracellular vesicles used in photo-assisted cancer therapy.

Engineering level	Photosensititizers	Moment of PS loading	Strategy mechanism	Main advantages	Limitations	Representative examples	Ref.
I. Cargo engineering							
Cargo Loading (Passive)	Ce6; ZnPc; Ru(tpy)(biq)2+; IR820; quercetin; fullerene derivatives	Post EV isolation	Simple incubation; hydrophobic PS partition into the EV lipid bilayer via hydrophobic interactions or via an exchange reaction.	Preserves membrane integrity and biological function; simple and cost-effective.	Low and variable loading efficiency; limited primarily to hydrophobic molecules.	Ce6@GEV (Goat milk EVs); EV-ZnPc; TEXs-tuning nanoassembly (TEXT) coloaded with biguanides and photosensitizers; IR820-TPE-Loaded Exosomes; C60/EVs	(81, 28, 82–84)
Cargo Loading (Active)	Hypericin; aggregation-induced emission luminogens; NIR-II organic small molecule; IR780; Pyropheophorbide-α; indocyanine Green; 4-(2-(5-(4-(diphenylamino)phenyl)thiophen-2-yl)vinyl)-1-methylquinolin-1-ium Iodide; Ir(III) complexes	Post EV isolation	Physical disruption (Sonication, Electroporation, Extrusion or freeze-thaw cycle) to create transient pores for cargo entry	High loading efficiency; enables encapsulation of diverse molecules (e.g., proteins, nucleic acids)	Potential membrane damage or EV aggregation; risk of degrading sensitive biological cargo	sEV-HYP; AIE@SDNV; MYM@iRGD-Exo; Sp-Exo/AI; M1@PAP; ExoTPP	(85, 86, 71, 30, 87, 31, 32, 88, 70)
Endogenous loading (donor cell pre-incubation)	Rose Bengal, chlorophyll-based carbon dots; anthraquinone; 5-ALA	Pre EV isolation	Donor cells are pre-incubated with a photosensitizer or photoactive precursor, allowing endogenous cargo loading or intracellular biosynthesis of photoactive metabolites that are subsequently incorporated into EVs during biogenesis.	Preserves EV membrane integrity and biological functionality; enables physiological cargo incorporation with minimal post-isolation manipulation.	Generally low and variable loading efficiency; cargo loading depends on donor cell uptake and intracellular sorting mechanisms; limited control over final cargo content.	CD@EXOMSC; PDEVs; RB-EVs	(25, 89, 90, 72)
II. Membrane engineering							
Chemical membrane engineering	Ce6, ICG, IR820	Post EV isolation	Surface functionalization via lipid anchoring (DSPE-PEG-Ligand) or Click Chemistry (DBCO-Azide)	Rapid and robust active targeting (e.g., folate, biotin); prolonged blood circulation time	Complex optimization; chemical modifications may interfere with protein-protein recognition.	IR820-TPE@B-Exo (Biotinylated); DBCO-Ce6 click-labeled EVs	(91, 84)
Ligand-mediated membrane engineering	PpIX, MYM, Ce6	Post EV isolation	Surface functionalization with tumor-targeting ligands, including peptides (e.g., iRGD, NLS-containing chimeric peptides) and aptamers (e.g., AS1411), to enhance receptor-specific recognition, deep tumor penetration, blood-brain barrier crossing, or subcellular targeting.	Improves targeting specificity, tissue penetration, BBB transport, and intracellular localization while increasing therapeutic efficacy and minimizing off-target effects.	Requires ligand synthesis/conjugation; ligand orientation and stability may influence targeting efficiency; increased manufacturing complexity.	ChiP-Exo; MYM@iRGD-Exo; AS1411-functionalized EVs.	(47, 77, 31)
Bioorthogonal membrane programming	DBCO-Chlorin e6 (DBCO-Ce6)	Post-EV isolation/Sequential targeting	Metabolic engineering generates artificial azide receptors on tumor cells, which are propagated intercellularly by EVs. Subsequent *in vivo* bioorthogonal click chemistry selectively anchors DBCO-functionalized photosensitizers to engineered tumor cells.	Overcomes receptor heterogeneity; enhances tumor accumulation and deep tissue penetration; enables secondary targeting and amplification of PDT efficacy.	Requires metabolic glycoengineering, sequential administration, and bioorthogonal click chemistry; increased therapeutic complexity.	Az-NPs/DBCO-Ce6	(91)
Stimuli-responsive membrane engineering	Various PS	Post-isolation	ROS-, pH- or enzyme-responsive membrane modifications exposing ligands or triggering release within tumors.	On-demand activation; reduced off-target toxicity.	Complex synthesis and validation.	HMSNs-GOx-Ce6@PFC-CPPO@C; Fe-G@DFs	(79, 26, 92)
Hybrid membrane engineering	Ce6, IR780, AIEgens	Post-isolation	Fusion of EVs with liposomes or biomimetic membranes.	Combines high loading capacity with biological targeting.	Fusion heterogeneity; difficult standardization.	Hybrid EV-liposome platforms	(24, 93, 79, 94)
III. Biological interface programming							
Genetic Engineering (Donor Cell Programming)	Rose Bengal (RB), Chlorin e6 (Ce6), other photosensitizers	During EV biogenesis (endogenous loading) or post-isolation	Donor cells are genetically engineered to express membrane proteins or targeting molecules (e.g., CARs, antibodies, nanobodies, affibodies, or CD47), enabling the secretion of EVs displaying correctly oriented functional ligands together with endogenous therapeutic cargo.	Ensures physiological membrane protein orientation, highly specific molecular recognition, prolonged circulation through immune evasion, multifunctional biological activity, and integration of targeting with intrinsic cellular functions.	Requires stable genetic modification of donor cells, complex manufacturing and quality control, limited production yield, and challenges for large-scale GMP production and regulatory approval.	Engineering CAR-NK Cell-Derived Exosome; Genetically Engineered Plasma Membrane Nanovesicles (GNVs) expressing antibodies, nanobodies or affibodies; CD47-overexpressing extracellular vesicles for immune-evasive photo-assisted therapy.	(74, 73, 27)
Tumor Microenvironment Programming	Ce6, CPPO, Dox-EMCH, AQ4N	During EV biogenesis and/or post-isolation	Immune cell-derived EVs or engineered biomimetic vesicles are designed to actively remodel the tumor microenvironment by repolarizing tumor-associated macrophages, relieving hypoxia, promoting dendritic cell activation, and enhancing antitumor immune responses in combination with photo-assisted therapy.	Transforms immunosuppressive TME into an immunostimulatory environment; synergizes with PDT-induced ICD; promotes systemic antitumor immunity and long-term therapeutic efficacy.	High biological complexity; donor cell-dependent variability; challenging mechanistic validation and translational standardization.	M1-PEV; M1-EVs; M1@Ce6-EVs	(80, 28, 95)
IV. Functional programming							
Multimodal therapeutic programming	RB, Ce6, IR820-TPE, MYM	Variable	Integration of PDT with PTT, SDT, chemotherapy, ferroptosis, immunotherapy and gene therapy.	Synergistic therapeutic responses.	Increased platform complexity.	IR820-TPE@B-Exo; MYM@iRGD-Exo	(84, 31)
Theranostic programming	AIEgens, IR820, SPIONs	Variable	Simultaneous imaging and therapy through fluorescence, photoacoustic imaging or MRI-guided PDT.	Image-guided therapy; treatment monitoring.	Multifunctional optimization required.	MYM@iRGD-Exo; IR820-TPE@B-Exo	(84, 31)

AIE, aggregation-induced emission luminogen; BBB, blood–brain barrier; CAR, chimeric antigen receptor; Ce6, chlorin e6; CL-PDT, Cerenkov luminescence photodynamic therapy; EVs, extracellular vesicles; FA, folic acid; FLI, fluorescence imaging; GEVs, goat milk-derived extracellular vesicles; ICD, immunogenic cell death; MPS, mononuclear phagocyte system; NIR, near-infrared; PDT, photodynamic therapy; PTT, photothermal therapy; ROS, reactive oxygen species; SDNVs, spinach-derived nanovesicles; SPDT, sono-photodynamic therapy; TAMs, tumor-associated macrophages; TEXs, tumor-derived extracellular vesicles. Engineering levels are organized according to the hierarchical framework proposed in this review, progressing from cargo engineering to membrane engineering, biological interface programming, and functional programming.

## 5 Functional evolution of EV-based photo-assisted therapeutic platforms

The engineering strategies described above have generated extracellular vesicle-based photo-assisted platforms with markedly different levels of biological and therapeutic complexity (**Figure 4**). To facilitate comparison among these systems, we propose a functional classification based on the principal role performed by the extracellular vesicle platform within the therapeutic process. Importantly, this framework should not be interpreted as a strict chronological sequence or as a hierarchy of therapeutic superiority. Instead, the proposed generations represent conceptual categories defined by the progressive acquisition of functions beyond PS transport, including selective targeting, integration of multiple therapeutic mechanisms, active modulation of tumor biology, and theranostic functionality. Classification relies on the predominant functional innovation of particular platforms, as they may amalgamate elements from multiple categories, rather than only on publication date or engineering intricacy.

**Figure 4:**
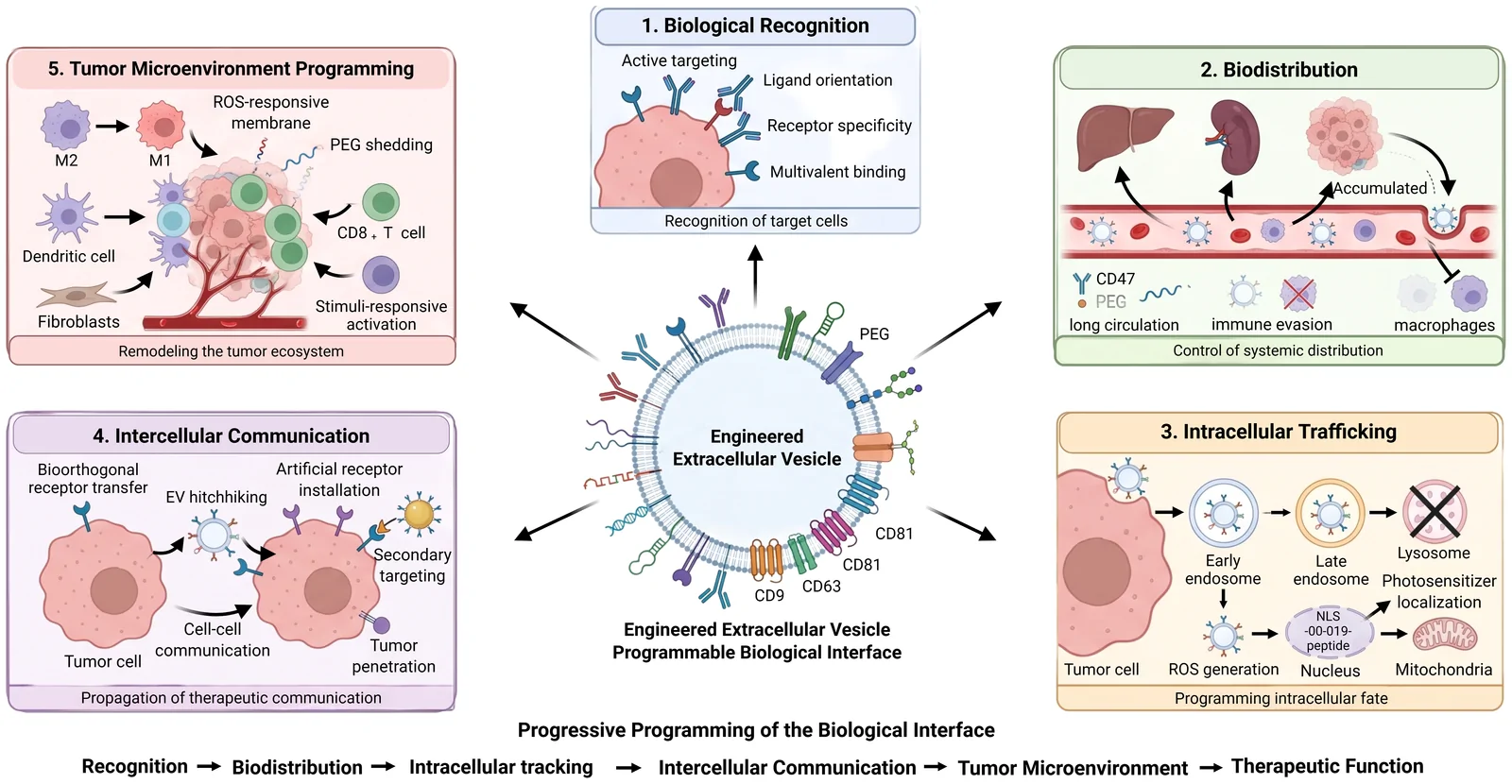
Engineering the extracellular vesicle biological interface to control therapeutic delivery and biological communication. The schematic illustrates five interconnected processes influenced by EV membrane engineering: biological recognition, biodistribution, intracellular trafficking, intercellular communication, and TME interactions. Representative engineering strategies can modulate EV behavior at different stages from systemic administration to cellular and microenvironmental interactions. These processes collectively determine the biological fate and therapeutic performance of engineered EVs. Created in BioRender.

Within this framework, five functional generations can be distinguished. First-generation platforms primarily exploit the intrinsic properties of native EVs to improve PS solubility, stability, cellular uptake, and tumor penetration. Second-generation systems introduce selective or biologically guided delivery through membrane functionalization, ligand-mediated recognition, genetic engineering, immune-evasion mechanisms, or homotypic targeting. Third-generation platforms integrate two or more complementary therapeutic modalities within a single extracellular vesicle-based system, such as photodynamic therapy combined with chemotherapy, photothermal therapy, sonodynamic therapy, metabolic therapy, or catalytic approaches. Fourth-generation platforms extend beyond drug delivery by actively modulating biological processes that influence therapeutic outcome, including antitumor immunity, tumor-associated macrophage polarization, hypoxia, tumor metabolism, immune checkpoints, and regulated cell death pathways such as ferroptosis. Fifth-generation platforms integrate therapy with diagnostic or image-guided capabilities, stimuli-responsive activation, spatiotemporal control, or other theranostic functions that enable simultaneous treatment localization, monitoring, and therapeutic intervention. These criteria provide the organizational basis for Sections 5.1–5.5 and are summarized in **Table 2**.

**Table 2:** Representative extracellular vesicle-based platforms supporting the proposed functional evolution of photo-assisted cancer therapy.

Functional generation	Defining criterion	Representative platform	Engineering innovation	Photo-assisted modality	Key biological function	Representative cancer model	Validation	Reference
First-generation	Improved PS delivery using intrinsic EV properties	mTHPC-EVs	Passive loading of clinical photosensitizer into endothelial EVs	PDT	Improved stability, tumor penetration and intracellular delivery	Multicellular tumor spheroids	*In vitro*	(10)
Second-generation	Selective or biologically guided targeting	TEXT	Hyaluronic acid-mediated self-assembly of tumor-derived EVs	PDT + Immunotherapy	Conversion of immunosuppressive TEX into immune-activating nanotherapeutics	4T1, B16F10	*In vivo*	(96)
Second-generation	Selective or biologically guided targeting	ChiP-Exo	Chimeric peptide membrane engineering	Dual-stage PDT	Sequential plasma membrane → nucleus targeting	HeLa, 4T1	Both	(47)
Second-generation	Selective or biologically guided targeting	CAR-NK Exosome Nanobombs	CAR genetic engineering + T7 peptide	PDT + Ferroptosis	HER2-directed BBB crossing and immune targeting	HER2+ brain metastasis	Both	(27)
Second-generation	Selective or biologically guided targeting	Er/RB@ExosCD47	CD47 donor-cell engineering	PDT + Ferroptosis	Immune evasion with prolonged circulation	Hepatocellular carcinoma	Both	(73)
Third-generation	Integration of complementary therapeutic modalities	HAL/3BP@X-MP	Homotypic microparticle engineering	PDT + Metabolic therapy	Mitochondrial metabolic reprogramming	4T1, CT26	Both	(79)
Third-generation	Integration of complementary therapeutic modalities	Ce6/GW4869@BMSC-EVs	Dual drug loading	PDT + Immunotherapy	Blocking endogenous EV secretion to potentiate ICD	TNBC	Both	(69)
Third-generation	Integration of complementary therapeutic modalities	PPA-Arg9/siPD-L1@M1-EVs	Immune-cell membrane engineering	PDT + NO + Immunotherapy	ECM remodeling, TAM repolarization and CD8 activation	Pancreatic cancer	*In vivo*	(80)
Third-generation	Integration of complementary therapeutic modalities	IHEL	Hybrid exosome-liposome fusion	PDT + PTT + Ferroptosis	Hypoxia relief and ferroptosis amplification	Melanoma	Both	(88)
Fourth-generation	Active modulation of tumor biology, TME, or regulated cell death	AIE@SDNV	Photosynthetic engineering	PDT + Immunotherapy	Oxygen self-generation and cGAS-STING activation	Breast cancer	Both	(30)
Fourth-generation	Active modulation of tumor biology, TME, or regulated cell death	Ce6@GEV	Cerenkov-mediated internal activation	CL-PDT	Depth-independent photodynamic therapy	Breast cancer	Both	(81)
Fifth-generation	Integration of imaging, monitoring, or spatiotemporal therapeutic control	ATO/IR780@SPION-Exo	Magnetic membrane engineering	PDT + Ferroptosis + Magnetic targeting	Image-guided BBB targeting and ferroptosis	Orthotopic GBM	Both	(32)
Fifth-generation	Integration of imaging, monitoring, or spatiotemporal therapeutic control	IR820-TPE@B-Exo	Heavy atom-free engineered EVs	Sono-photodynamic therapy	Heavy atom-free multimodal theranostics	Solid tumors	Both	(84)
Fifth-generation	Integration of imaging, monitoring, or spatiotemporal therapeutic control	MYM@iRGD-Exo	Peptide engineering + NIR-II imaging	FLI/PTI/PDT/PTT	NIR-II image-guided intelligent multimodal therapy	Orthotopic GBM	Both	(31)

BBB, blood–brain barrier; CAR, chimeric antigen receptor; CL-PDT, Cerenkov luminescence photodynamic therapy; ECM, extracellular matrix; EVs, extracellular vesicles; FLI, fluorescence imaging; GBM, glioblastoma; ICD, immunogenic cell death; NIR-II, second near-infrared window; PDT, photodynamic therapy; PTT, photothermal therapy; ROS, reactive oxygen species; SPDT, sono-photodynamic therapy; TAMs, tumor-associated macrophages; TEXs, tumor-derived extracellular vesicles. The functional generations proposed here represent a conceptual framework developed in this Review and should not be interpreted as strict chronological stages or as a hierarchy of therapeutic superiority. Platforms are assigned according to their dominant functional innovation, although individual systems may exhibit characteristics of more than one generation. Representative examples were selected to illustrate each category rather than to provide an exhaustive survey of reported extracellular vesicle-based photo-assisted systems.

### 5.1 First-generation platforms: EV-mediated photosensitizer delivery

The earliest applications of EVs in photo-assisted therapy were primarily focused on overcoming the physicochemical limitations of conventional PSs. Many clinically relevant PSs (e.g., Ce6, ZnPc, or mTHPC) exhibit poor aqueous solubility, strong hydrophobicity, aggregation under physiological conditions, limited intracellular accumulation and heterogeneous tumor penetration. Initial studies therefore explored the ability of naturally secreted EVs to improve PS delivery while preserving their photodynamic activity.

A landmark contribution was provided by Fuhrmann et al., who demonstrated that porphyrin-loaded EVs significantly enhanced intracellular uptake and photodynamic cytotoxicity compared with free PS (23). Early comparative studies suggested that EV-mediated delivery can improve PS accumulation and penetration relative to liposomal formulations in specific experimental models. For example, in three-dimensional HT29 multicellular tumor spheroids, mTHPC-loaded EVs produced approximately 3.5-fold greater mTHPC accumulation than the liposomal formulation Foslip^®^ after 3 h of incubation and approximately 2-fold greater accumulation after 24 h. EV-mediated delivery also improved penetration toward the spheroid core, with detectable mTHPC fluorescence at depths of 100 μm where free and liposomal formulations showed only minimal signal (10). This efficiency is attributed to the presence of specific membrane proteins (tetraspanins, adhesion molecules) and a unique lipid composition that facilitates superior cellular uptake, often through caveolae-dependent endocytosis.

These pioneering studies established the biological rationale for EV-mediated PDT by demonstrating simultaneous improvements in drug stability, cellular internalization, and tumor penetration *in vitro* without extensive engineering modifications. At this stage, EVs primarily functioned as endogenous drug carriers that exploited their intrinsic biological properties to overcome pharmaceutical limitations associated with free PSs.

### 5.2 Second-generation platforms: targeted and biologically guided delivery

Once the feasibility of EV-mediated PS delivery had been established, engineering efforts rapidly shifted toward improving delivery specificity. The objective was no longer simply to transport PSs but to control their biodistribution, cellular recognition and accumulation within tumors.

Multiple membrane engineering strategies—including peptide decoration, aptamer conjugation, antibody functionalization, lipid insertion, bioorthogonal chemistry and genetic programming of parental cells—enabled increasingly selective targeting of tumor-associated receptors. Engineered EVs extend delivery beyond passive accumulation through the EPR effect by enabling active recognition of specific cellular populations, improved interaction with biological barriers, and modulation of immune clearance.

Particularly noteworthy are bioorthogonal receptor programming strategies, in which metabolically engineered EVs transfer artificial chemical receptors to recipient tumor cells, enabling subsequent rounds of highly selective nanoparticle targeting (24). Likewise, genetic engineering approaches involving CAR-NK-derived exosomes and CD47-overexpressing vesicles further expanded targeting specificity while simultaneously improving circulation time and immune compatibility (27, 73).

At this stage, EVs ceased to function merely as delivery vehicles and became biologically guided transport systems capable of dynamically interacting with recipient tissues.

### 5.3 Third-generation platforms: multimodal therapeutic integration

A major milestone in EV evolution has been the integration of multiple therapeutic modalities within a single biomimetic platform. Third-generation platforms expand the therapeutic payload beyond PSs by co-encapsulating chemotherapeutic agents, nucleic acids, immunomodulators, catalytic nanoparticles, or oxygen-generating systems.

Several platforms combine PDT with chemotherapy, significantly improving treatment efficacy by simultaneously inducing oxidative stress and DNA damage (97, 98). These platforms utilize light-triggered drug release mechanisms, where the ROS generated during PDT disrupt the EV membrane, facilitating the rapid release of chemotherapeutic agents directly within the tumor cell. Other strategies integrate PDT with PTT or SDT, thereby overcoming limitations associated with tissue penetration and tumor hypoxia (99, 100). More recently, catalytic nanozymes and oxygen-generating nanoparticles have been incorporated into EVs to alleviate hypoxia and sustain ROS production during photoactivation, one of the principal challenges limiting PDT in solid tumors. Engineered EVs have been loaded with perfluorocarbons (PFC) as oxygen reservoirs (71); or integrated with photosynthetic machinery from plant-derived nanovesicles (e.g., spinach-derived nanovesicles) to generate oxygen *in situ* upon light irradiation (30).

The development of multifunctional hybrid systems has further expanded these capabilities. Fusion between EVs and liposomes, polymeric nanoparticles, metal-organic frameworks or inorganic nanomaterials combines the biological communication properties of native EVs with the engineering versatility of synthetic nanotechnology, resulting in multifunctional biomimetic platforms capable of controlled drug release, multimodal imaging and combinatorial therapy.

These advances represent a transition from single-drug delivery systems toward integrated therapeutic platforms capable of orchestrating multiple complementary mechanisms within a single nanosystem.

### 5.4 Fourth-generation platforms: biological and tumor microenvironment programming

The most recent evolution of EV-based photo-assisted therapy extends well beyond improving drug delivery or enhancing tumor targeting. Engineered EVs are no longer viewed simply as delivery vehicles but as biological modulators capable of coordinating immune responses, remodeling the TME, and directing specific forms of regulated cell death (RCD). Consequently, therapeutic efficacy is determined not only by the amount of PS delivered to the tumor but also by the biological pathways activated following photoactivation.

One of the most important discoveries supporting this transition is the realization that PDT can function as an immune-modulating treatment. In addition to producing ROS and direct oxidative damage, PDT induces ICD, a regulated process characterized by the exposure or release of damage-associated molecular patterns (DAMPs), including calreticulin (CRT), ATP and high-mobility group box 1 (HMGB1). These danger signals promote dendritic cell maturation, antigen presentation and subsequent activation of tumor-specific CD8^+^ cytotoxic T lymphocytes, thereby converting local phototoxicity into systemic antitumor immunity. However, although PDT possesses intrinsic immunogenic potential, the magnitude of this response is frequently limited by the highly immunosuppressive TME.

EVs have emerged as particularly attractive platforms for overcoming these immunological barriers. Owing to their natural role in intercellular communication, EVs can simultaneously deliver therapeutic cargoes while transferring membrane proteins, cytokines, nucleic acids and signaling molecules capable of modulating immune cell function. In these systems, engineered EVs can simultaneously transport therapeutic cargo and contribute biologically to PDT-induced immune activation.

Several studies have demonstrated that EVs derived from immune cells retain part of the biological functionality of their parental cells. For example, macrophage-derived EVs generated from M1-polarized macrophages inherit pro-inflammatory membrane proteins and cytokine-associated signaling pathways capable of promoting macrophage repolarization within tumors, thereby reversing the immunosuppressive phenotype commonly associated with tumor-associated macrophages (28, 80, 87). Similarly, dendritic cell-derived EVs enhance antigen presentation and T-cell priming, whereas NK-cell-derived EVs preserve intrinsic cytotoxic activity and can directly eliminate tumor cells while simultaneously potentiating PDT-induced immune responses. More recently, genetically engineered CAR-NK-derived exosomes have demonstrated highly selective recognition of HER2-positive tumors while integrating receptor-mediated targeting with immune-mediated tumor killing, illustrating how EV engineering can combine targeted delivery and immunotherapy within a single platform (27).

Beyond immune-cell-derived vesicles, membrane engineering strategies increasingly seek to manipulate the biological dialog established between tumors and their surrounding microenvironment. Some engineered EV platforms have additionally been designed to modify macrophage polarization, stimulate dendritic-cell maturation, promote T-cell infiltration, reduce immune-checkpoint signaling, and remodel stromal interactions. These observations indicate that EV-mediated photo-assisted therapy is progressively evolving toward an integrated form of photoimmunotherapy in which oxidative damage and immune activation become inseparable therapeutic processes.

In parallel with immune programming, recent studies have demonstrated that EVs can also direct RCD pathways activated following photoirradiation. Traditionally, the cytotoxic effects of PDT were attributed primarily to apoptosis or necrosis induced by ROS-mediated oxidative damage. It is now evident, however, that ROS production constitutes only the initiating event, whereas the ultimate biological outcome depends on the intracellular signaling pathways activated after oxidative stress (101). Consequently, modern EV-based platforms increasingly aim to direct photo-induced oxidative damage toward specific forms of RCD that maximize therapeutic efficacy while simultaneously promoting antitumor immunity.

Among these pathways, ferroptosis has emerged as the most extensively investigated and clinically promising. Ferroptosis is characterized by iron-dependent lipid peroxidation resulting from the failure of cellular antioxidant defense systems, particularly the glutathione (GSH)/glutathione peroxidase 4 (GPX4) axis (102). Because PDT produces abundant ROS that readily initiate phospholipid oxidation, PDT provides an ideal biochemical environment for ferroptosis induction. Nevertheless, PDT alone is often insufficient to sustain ferroptotic cell death because tumor cells rapidly restore redox homeostasis through GPX4 activity, glutathione regeneration and other antioxidant pathways (3).

EVs offer an exceptional opportunity to overcome these limitations by enabling the simultaneous delivery of PSs together with ferroptosis-promoting agents. Recent multifunctional platforms have incorporated iron-containing nanoparticles, catalytic nanozymes, GSH-depleting compounds, GPX4 inhibitors, system Xc^-^ inhibitors, ferroptosis-related siRNAs and oxygen-generating nanomaterials within engineered EVs (32, 73, 85, 88). By combining enhanced ROS production with suppression of antioxidant defenses, these systems markedly amplify lipid peroxidation and overcome resistance mechanisms that frequently limit conventional PDT. Importantly, ferroptosis itself has recently been shown to reinforce antitumor immunity through increased release of immunostimulatory mediators and improved antigen presentation, suggesting that EV-mediated photo-ferrotherapy may establish a positive feedback loop between oxidative stress, RCD and immune activation.

Although ferroptosis currently represents the most advanced application of RCD-oriented photo-assisted therapy, other RCD pathways—including pyroptosis, necroptosis and cuproptosis—are beginning to attract increasing attention (53). While evidence supporting their integration into EV-mediated PDT remains limited, these pathways illustrate the growing interest in exploiting engineered extracellular vesicles not merely to deliver cytotoxic agents but to selectively orchestrate the molecular mechanisms that ultimately determine cancer cell fate.

Collectively, these advances illustrate one of the most significant conceptual transformations in the field of photo-assisted nanomedicine. Collectively, these advances expand the role of engineered EVs toward biological regulation through the integration of immune modulation, TME remodeling, and regulated cell death. This convergence of photoimmunotherapy and RCD-oriented nanomedicine represents the current frontier of EV engineering and is expected to play a central role in the development of next-generation precision phototherapies.

### 5.5 Fifth-generation platforms: theranostic and image-guided systems

The latest stage in the evolution of EV-based photo-assisted therapy is characterized by the convergence of therapeutic delivery, molecular imaging, and precision medicine into integrated theranostic platforms. Unlike previous generations, which primarily focused on improving therapeutic efficacy, these advanced systems are designed to simultaneously diagnose, monitor, and treat tumors while preserving the intrinsic biological advantages of EV-mediated delivery. These platforms extend EV-based therapy by integrating therapeutic delivery with imaging, treatment localization, or monitoring capabilities, thereby supporting image-guided and theranostic applications (103).

The intrinsic biological properties of EVs—including their endogenous membrane composition, high biocompatibility, efficient cellular internalization, and ability to transport therapeutic and imaging cargoes across biological barriers—make them particularly attractive as theranostic platforms. These properties facilitate not only targeted drug delivery but also the incorporation of diagnostic probes that enable real-time monitoring of biodistribution, tumor accumulation, and treatment response (15, 104).

Recent engineering strategies have incorporated a wide variety of imaging agents into EVs, including fluorescent dyes, aggregation-induced emission (AIE) luminogens, near-infrared (NIR-I and NIR-II) fluorophores, photoacoustic contrast agents, magnetic nanoparticles, radionuclides, and multimodal imaging probes. These multifunctional systems enable simultaneous visualization of EV biodistribution, intracellular trafficking, therapeutic activation, and treatment efficacy while maintaining the biological advantages of EV-mediated delivery (31, 105, 106).

Among optical imaging modalities, NIR-II fluorescence imaging has emerged as one of the most promising technologies because it provides deeper tissue penetration, higher spatial resolution, and reduced tissue autofluorescence compared with conventional visible or NIR-I imaging. A representative example is the recently reported MYM@iRGD-Exo platform, in which an NIR-II phototheranostic molecule was encapsulated within engineered exosomes and functionalized with the tumor-penetrating peptide iRGD. This system efficiently crossed the blood-brain barrier, accumulated within orthotopic glioblastoma, enabled NIR-II fluorescence imaging, and simultaneously mediated photodynamic and photothermal therapy while promoting T-cell infiltration and antitumor immune activation (31).

Aggregation-induced emission (AIE)-based PSs have further expanded the capabilities of EV-mediated theranostics. Unlike conventional fluorophores, which often suffer from aggregation-caused quenching, AIE luminogens become highly fluorescent upon aggregation while simultaneously exhibiting excellent ROS generation. Engineered exosomes carrying AIE PSs therefore integrate fluorescence imaging with efficient photodynamic and photothermal therapy, enabling real-time visualization of therapeutic delivery without compromising photoactivity (84, 107).

Photoacoustic imaging has likewise become increasingly integrated into EV-based theranostic systems. By combining the high optical contrast of fluorescence imaging with the penetration depth and spatial resolution of ultrasound, photoacoustic-guided EV platforms allow non-invasive monitoring of tumor oxygenation, nanoparticle accumulation, and therapeutic activation. For example, Jang et al. developed an exosome-based platform integrating photoacoustic imaging with PDT and immunotherapy for pancreatic cancer, enabling image-guided treatment while simultaneously enhancing antitumor immune responses (108).

Magnetic resonance imaging (MRI) has also been successfully incorporated into EV-mediated phototheranostics. Early studies demonstrated the feasibility of generating EVs simultaneously loaded with the clinical PS Foscan and iron oxide nanoparticles, allowing concurrent PDT, MRI, magnetic manipulation, and hyperthermia within a single biomimetic platform (15). These studies established one of the earliest demonstrations that engineered EVs could integrate diagnostic imaging and therapy while preserving the biological characteristics of endogenous vesicles.

The evolution of EV-based theranostics has subsequently progressed toward multimodal imaging, in which two or more complementary imaging techniques are integrated into a single nanosystem. Hybrid platforms combining fluorescence imaging with MRI, photoacoustic imaging, or magnetic guidance provide synergistic diagnostic information by simultaneously exploiting the high sensitivity of optical imaging and the anatomical or functional information provided by tomographic modalities. Such multimodal approaches improve treatment planning, facilitate longitudinal monitoring of therapeutic response, and may accelerate clinical translation (103).

Importantly, contemporary theranostic systems extend beyond the incorporation of imaging agents. Engineering strategies increasingly exploit stimuli-responsive biological interfaces capable of activating therapeutic functions only after exposure to tumor-associated biochemical or physical signals, including acidic pH, ROS, hypoxia, GSH concentration, enzymatic activity, or external irradiation. These responsive systems enable spatially and temporally controlled therapeutic activation while reducing off-target toxicity, thereby integrating molecular diagnosis with precision therapy.

The rapid evolution of EV-based photo-assisted therapy has generated a remarkable diversity of multifunctional biomimetic platforms that progressively integrate targeting, microenvironment modulation, multimodal therapeutic mechanisms, and image-guided treatment within a single nanosystem. **Table 2** summarizes representative EV-based photo-assisted platforms reported to date, highlighting their engineering strategies, therapeutic modalities, and the key biological innovations that have driven the functional evolution of the field.

## 6 Challenges and barriers for clinical translation

Despite the rapid expansion and increasing sophistication of EV-based photo-assisted therapeutic platforms at the preclinical level, their clinical translation remains at a very early stage (109). To date, no EV-based platform integrating a PS or other photo-/sono-responsive therapeutic cargo for cancer treatment has reached clinical evaluation in humans. Thus, the evidence supporting EV-mediated PDT, PTT, SDT, photoimmunotherapy, and related multimodal strategies remains predominantly preclinical. This translational gap is particularly relevant given the increasing complexity of engineered EV systems, as platforms combining targeting ligands, multiple therapeutic cargos, genetic modifications, inorganic nanomaterials, or stimuli-responsive functions introduce additional challenges in manufacturing, characterization, safety assessment, and regulatory classification.

From a translational perspective, not all current limitations have equivalent priority. Before EV-based photo-assisted platforms can realistically progress toward first-in-human studies, at least five interrelated challenges must be addressed: (i) definition of a reproducible therapeutic product with clearly established critical quality attributes; (ii) scalable and GMP-compatible production, purification, and storage; (iii) standardized determination of therapeutic dose and potency; (iv) rigorous characterization of biodistribution, pharmacokinetics, and short- and long-term safety; and (v) establishment of a regulatory strategy compatible with the increasing complexity of engineered EV products. These requirements become progressively more demanding as EV platforms incorporate targeting ligands, genetic modifications, multiple therapeutic cargos, inorganic components, or stimuli-responsive functions. Consequently, engineering sophistication should be evaluated not only in terms of therapeutic performance but also according to whether the resulting product can be reproducibly manufactured, characterized, dosed, and evaluated under clinically relevant conditions.

Importantly, the absence of clinical trials specifically evaluating EV-based photo-assisted therapy should be distinguished from the broader clinical development of EV therapeutics (**Table 3**). EV-based interventions have entered early-phase clinical studies in oncology and other diseases, including dendritic cell-derived exosome vaccines and engineered EVs carrying therapeutic nucleic acids or small molecules. These studies provide important proof that EV-based products can be manufactured and administered clinically, but they also highlight the substantial gap between relatively defined EV therapeutics and the increasingly complex multifunctional systems currently being developed for photo-assisted cancer therapy.

**Table 3:** Representative clinical trials evaluating extracellular vesicle-based therapeutic strategies in oncology.

Trial ID	EV source/platform	Therapeutic strategy	Indication	Phase	Status	Photo-assisted
NCT01159288	Dendritic cell-derived exosomes	Antigen-loaded exosome vaccine (Dex2)	NSCLC	Phase II	Completed	No
NCT03608631	MSC-derived exosomes	KRAS^G12D siRNA delivery	Pancreatic adenocarcinoma	Phase I - II	Active/recruiting	No
NCT01294072	Plant-derived vesicles	Curcumin delivery	Colon cancer	Early clinical study	Unknown status	No
NCT05375604	Engineered exosomes	Oligonucleotide delivery	Advanced solid tumors with liver involvement	Phase I	Terminated	No

No documented clinical trial assessing EV-based PDT, PTT, SDT, or alternative photo-assisted cancer therapies was found during the paper revision. The studies presented here serve as illustrative instances of therapeutic EV-based interventions and are provided to contextualize the present translational progress of the field rather than to offer a comprehensive overview of EV clinical trials.

One of the major biological challenges arises from the intrinsic heterogeneity of EV. Unlike synthetic nanoparticles, which can be manufactured with relatively homogeneous physicochemical properties, EVs constitute highly heterogeneous populations that vary in size, membrane composition, lipid content, protein repertoire, nucleic acid cargo, and biological activity according to their cellular origin, physiological state, and isolation procedure (110). Even EVs secreted by the same cell type may exhibit substantial batch-to-batch variability depending on culture conditions, passage number, cellular activation status, and environmental stimuli (111, 112). This biological complexity complicates the establishment of standardized manufacturing protocols and makes direct comparisons among studies particularly difficult. Furthermore, the absence of universally accepted criteria for defining EV subpopulations continues to hamper reproducibility across laboratories despite the recommendations proposed by the International Society for Extracellular Vesicles (ISEV) through the MISEV guidelines (113).

In this sense, the first requirement is the definition of the EV therapeutic product itself. EV preparations contain heterogeneous vesicle populations whose composition and biological activity can vary with donor-cell source, culture conditions, isolation procedure, and production batch. For engineered EVs, additional variability may arise from cargo-loading efficiency, surface modification, or genetic manipulation of producer cells. Clinical translation will therefore require the identification of critical quality attributes capable of linking EV identity and composition to therapeutic function, together with release criteria that ensure batch-to-batch consistency. This represents a particularly important challenge for multifunctional photo-assisted systems, in which both the EV component and the photoactive cargo contribute to therapeutic activity.

A second major challenge concerns large-scale manufacturing and standardization. Most experimental studies employ ultracentrifugation, density gradient separation, precipitation methods, size-exclusion chromatography (SEC), tangential flow filtration (TFF), or combinations of these techniques for EV isolation. Although these approaches provide acceptable purity for laboratory research, they differ considerably in recovery yield, scalability, reproducibility, vesicle integrity, and contaminant removal (114). Consequently, isolation methodology itself may substantially influence therapeutic efficacy by altering EV composition and biological activity. The development of Good Manufacturing Practice (GMP)-compliant production workflows therefore remains one of the principal priorities for clinical translation. Emerging technologies such as continuous tangential flow filtration, microfluidic isolation platforms, affinity-based purification, and automated manufacturing systems are expected to improve scalability while maintaining product consistency, although these approaches still require extensive validation under industrial conditions (115–117). Translation will require scalable producer-cell expansion, chemically defined culture conditions, high-throughput EV isolation and purification, removal of process-related contaminants, validated storage conditions, and preservation of biological activity throughout manufacturing. Importantly, increases in production yield cannot be considered independently from product quality, because changes in culture conditions or bioprocessing parameters may alter EV composition and potency. Manufacturing strategies must therefore optimize yield and reproducibility simultaneously rather than maximizing vesicle production alone.

Efficient cargo loading represents another important technological bottleneck. Although numerous engineering strategies—including passive incubation, electroporation, sonication, extrusion, freeze-thaw cycling, membrane permeabilization, endogenous cellular loading, and more recently remote-loading approaches such as SEAL—have been developed to increase encapsulation efficiency, no single methodology has yet demonstrated universal applicability across the wide diversity of therapeutic payloads employed in photo-assisted therapy. High loading efficiency frequently occurs at the expense of membrane integrity, vesicle stability, or biological activity, whereas gentler loading methods often exhibit poor encapsulation efficiency. Consequently, future engineering efforts will likely require cargo-specific optimization strategies capable of maximizing loading efficiency while preserving the intrinsic biological characteristics that distinguish EVs from synthetic nanocarriers.

Equally important are the challenges associated with membrane engineering and biological functionality. Increasingly sophisticated membrane modifications have enabled remarkable improvements in tumor targeting, immune modulation, and intercellular communication. However, every additional engineering step increases manufacturing complexity and may alter the native biological behavior of EVs. Surface conjugation, membrane fusion, genetic programming of donor cells, hybrid nanostructure formation, and stimuli-responsive modifications all introduce additional variables that require rigorous physicochemical characterization and biological validation.

Safety and immunogenicity represent additional considerations as EV engineering becomes increasingly complex. Although native EVs are generally regarded as biocompatible, their immunological behavior depends on multiple factors, including cellular origin, surface composition, endogenous and exogenous cargo, dose, administration schedule, and manufacturing conditions. In particular, EVs derived from allogeneic or immune cells may retain immunologically active membrane proteins, including major histocompatibility complex molecules, whereas genetic modification of donor cells or the incorporation of non-native targeting ligands, receptors, synthetic components, or therapeutic cargos may introduce additional antigenic determinants or alter EV recognition and clearance by the immune system. This issue is particularly relevant for platforms derived from genetically engineered immune cells, such as CAR-NK EVs, or donor cells modified to express surface proteins such as CD47. Importantly, the preclinical photo-assisted platforms discussed in this review have generally reported favorable short-term tolerability rather than overt treatment-related immunotoxicity; for example, CD47-engineered exosomes carrying erastin and Rose Bengal showed reduced hepatic toxicity and no evident major liver or kidney toxicity in treated mice (73). Nevertheless, the majority of research was primarily structured to evaluate antitumor effectiveness and traditional systemic toxicity instead of focusing on EV-specific immunogenicity, the generation of anti-EV antibodies, complement activation, cytokine responses, or the implications of repeated administration. Thus, the current absence of reported severe immune adverse effects should not be interpreted as evidence of immunological neutrality. Translation of extensively engineered EVs will require dedicated immunotoxicity studies addressing both acute and repeated-dose exposure, including innate and adaptive immune activation, complement responses, anti-EV antibodies, cytokine release, altered biodistribution, and potential immune consequences of donor-cell-derived and engineered membrane components (109, 118, 119).

The *in vivo* pharmacokinetics and biodistribution of engineered EVs also remain incompletely characterized. Although EVs generally exhibit superior biocompatibility compared with many synthetic nanocarriers, systemic administration frequently results in rapid accumulation within the liver, spleen, lungs, and other organs of the mononuclear phagocyte system (120, 121). While membrane engineering strategies such as CD47 overexpression, PEGylation, active targeting ligands, and cell-specific membrane programming have partially improved circulation time and tumor accumulation, quantitative biodistribution studies remain surprisingly scarce. Importantly, many preclinical investigations rely primarily on fluorescence imaging, which may overestimate tumor accumulation because fluorescent labels do not necessarily reflect the actual biodistribution of intact vesicles or their therapeutic cargoes. Future studies should therefore incorporate quantitative imaging methodologies, isotope labeling, elemental analysis, or complementary pharmacokinetic approaches capable of accurately determining EV fate following systemic administration.

Dose definition and potency assessment represent another critical translational gap. EV preparations are commonly normalized according to particle number, protein concentration, or parental-cell equivalents, yet these parameters do not necessarily predict biological activity. This issue becomes even more complex for photo-assisted platforms because therapeutic efficacy depends simultaneously on EV dose, photoactive cargo content, loading efficiency, light or ultrasound exposure, and the biological activity of the engineered vesicle. Clinically relevant development will therefore require validated potency assays that reflect the proposed mechanism of action and enable reproducible comparison among manufacturing batches. For multifunctional systems, defining which component constitutes the principal active element—and which analytical parameter best predicts therapeutic activity—will be essential for dose selection and regulatory evaluation.

An additional layer of complexity is imposed by the optical limitations associated with light delivery. While EV engineering can substantially improve PS accumulation within tumors, therapeutic efficacy remains fundamentally dependent on adequate light penetration and tissue oxygenation regarding PDT. Deep-seated tumors continue to represent a major challenge for conventional PDT owing to limited penetration of visible light and the oxygen consumption associated with ROS generation. Recent developments involving NIR-II photoactivation, sonodynamic therapy, oxygen-generating nanoplatforms, X-ray-activated scintillating nanoparticles, and multimodal photo-assisted systems have partially addressed these limitations, yet further optimization will be necessary before these technologies become broadly applicable in clinical oncology.

Regulatory considerations represent another critical barrier. Engineered EVs occupy a unique position at the interface between biological therapeutics, nanomedicine, advanced therapy medicinal products (ATMP), and combination products. Consequently, current regulatory frameworks developed for conventional pharmaceuticals or synthetic nanomaterials are often insufficient to address the complexity of EV-based therapeutics. Existing FDA and EMA pathways therefore rely on adapted biologics or ATMP frameworks rather than EV-specific rules. Standardized criteria regarding characterization, potency assays, batch release, stability testing, sterility, storage conditions, and quality control remain under active development. Harmonization among regulatory agencies, together with the establishment of internationally accepted manufacturing standards, will therefore be essential for successful clinical implementation (122).

Finally, although the preclinical evidence supporting EV-based photo-assisted therapy has expanded dramatically during recent years, clinical evidence remains remarkably limited. Most studies have been performed in small animal models using xenograft or syngeneic tumor systems that only partially reproduce the biological complexity of human cancer. Well-designed translational studies incorporating clinically relevant tumor models, standardized manufacturing protocols, long-term safety evaluation, pharmacokinetic characterization, and rigorous comparison with existing therapeutic alternatives remain urgently needed. Moreover, direct comparisons between engineered EVs and clinically approved nanomedicines are still relatively uncommon, making it difficult to determine the true added value of biomimetic delivery systems under clinically relevant conditions.

Taken together, these challenges highlight that the future success of extracellular vesicle-based photo-assisted therapy will depend not only on further advances in nanotechnology but also on improvements in standardization, manufacturing, regulatory science, and translational methodology. The extraordinary biological versatility of EVs has already transformed the conceptual landscape of photo-assisted nanomedicine. However, realizing their full clinical potential will require multidisciplinary efforts integrating cell biology, biomaterials science, pharmaceutical engineering, manufacturing technology, imaging, regulatory science, and clinical oncology. Successfully addressing these challenges will ultimately determine whether engineered extracellular vesicles evolve from highly promising experimental platforms into clinically approved precision therapeutics.

These considerations also highlight the importance of distinguishing current achievements from future expectations. At present, preclinical studies have demonstrated that EVs can transport photoactive agents, improve cellular delivery, incorporate targeting mechanisms, support multimodal therapeutic combinations, and modulate selected components of the TME. In parallel, the broader EV field has established that EV-based therapeutic products can be manufactured and administered to humans in early clinical studies. However, these achievements should not be extrapolated to the clinical readiness of engineered EV-based photo-assisted platforms. Reproducible large-scale manufacturing, standardized potency assays, clinically validated dosing, quantitative pharmacokinetics, repeated-dose safety, and regulatory pathways for multifunctional engineered EVs remain unresolved. Accordingly, concepts such as highly programmable, adaptive, or personalized EV-based photo-assisted systems should currently be regarded as future technological objectives rather than established clinical capabilities.

## 7 Future perspective

The translational barriers outlined above define the conditions under which future EV-based photo-assisted platforms will need to develop. The guidelines outlined in this segment should be viewed as potential prospects instead than established competencies that have attained clinical maturity.

The extraordinary progress achieved in EV engineering over the past decade has fundamentally reshaped the landscape of photo-assisted cancer therapy. As discussed throughout this review, engineered EVs have evolved from naturally occurring drug carriers into multifunctional biomimetic systems capable of integrating targeted delivery, molecular imaging, immune modulation, and regulated cell death within a single therapeutic platform. However, the next phase of development is unlikely to be driven solely by incremental improvements in cargo loading or membrane engineering. Instead, the future of EV-based photomedicine will increasingly depend on the ability to design adaptive, programmable, and clinically translatable biomimetic systems that respond dynamically to the biological complexity of individual tumors.

A highly promising avenue will be the shift from passive delivery systems to physiologically programmable nanoplatforms. Future EVs are anticipated to not only deliver therapeutic medicines but also to actively detect biochemical signals within the TME and react by regulating cargo release, sequentially activating therapeutic pathways, or dynamically adjusting intercellular communication. The advancement of stimuli-responsive membranes, bioorthogonal receptor transmission, EV hitchhiking, and programmable biological interfaces exemplifies the initial strides toward this novel generation of adaptive therapeutic systems. Future engineering strategies will likely integrate multiple biological feedback mechanisms capable of coordinating drug delivery, immune activation, and RCD according to the evolving characteristics of the TME.

Another important trend will be the increasing convergence between photo-assisted therapy and precision immuno-oncology. Growing evidence indicates that PDT should no longer be considered exclusively as a local cytotoxic treatment but rather as an initiator of systemic immune responses through ICD induction. Future EV platforms will therefore likely combine PSs with immune checkpoint inhibitors, cytokines, nucleic acid therapeutics, macrophage-polarizing molecules, dendritic cell activators, or engineered immune-cell-derived vesicles to maximize both local tumor eradication and long-term immunological memory. Such integrated photoimmunotherapeutic approaches may become particularly valuable for metastatic disease, where systemic immune activation is essential for durable clinical responses.

Among the various RCD pathways currently under investigation, ferroptosis is expected to occupy a central position in the next generation of EV-mediated photo-assisted therapies. The strong biochemical convergence between photodynamic ROS generation and ferroptotic lipid peroxidation creates unique opportunities for synergistic therapeutic design. Future platforms will likely incorporate increasingly sophisticated combinations of PSs, iron-containing nanomaterials, catalytic nanozymes, GSH-depleting agents, GPX4 inhibitors, and ferroptosis-regulating nucleic acids to amplify oxidative stress while overcoming intrinsic resistance mechanisms. At the same time, the interaction between ferroptosis and antitumor immunity represents an emerging research area with considerable translational potential. Expanding our understanding of how different RCD modalities interact with immune activation may ultimately enable the rational design of EVs capable of orchestrating multiple complementary therapeutic mechanisms within a single biomimetic platform.

The source of EVs is also expected to diversify considerably. Although mammalian cell-derived EVs currently dominate the field, increasing attention is being directed toward plant-derived extracellular vesicles (PDEVs), bacterial membrane vesicles, hybrid membrane systems, and biomimetic artificial EVs. PDEVs are particularly attractive because they offer excellent scalability, low production costs, reduced biosafety concerns, and intrinsic biological activities, including antioxidant and immunomodulatory properties. Their combination with photoactive nanomaterials, therapeutic nucleic acids, or immunomodulatory cargoes may provide highly accessible alternatives for large-scale clinical manufacturing while preserving many of the biological advantages associated with naturally derived vesicles. Likewise, hybrid systems generated by combining natural EV membranes with synthetic nanomaterials may further bridge the gap between biological functionality and engineering flexibility.

Future advances will also depend on improvements in manufacturing technologies. Automated bioreactor systems, continuous tangential flow filtration, affinity-based purification, microfluidic production platforms, and closed-system GMP manufacturing are expected to substantially improve reproducibility and scalability while reducing production costs. In parallel, standardized quality control methodologies integrating proteomics, lipidomics, transcriptomics, and advanced single-particle characterization techniques will facilitate more rigorous assessment of EV identity, potency, and batch consistency. Such technological advances will be essential for regulatory approval and widespread clinical implementation.

From a prospective perspective, computational biology, machine learning (ML), and artificial intelligence (AI) could eventually contribute to the rational design of engineered EV platforms by integrating multidimensional datasets related to EV composition, cargo loading, cellular interactions, biodistribution, and therapeutic response. Potential applications include the computational prioritization of targeting ligands, prediction of cargo–EV compatibility, modeling of biodistribution, optimization of formulation parameters, and integration of imaging and molecular data for treatment planning (123). However, these possibilities remain largely aspirational in the specific context of EV-based photo-assisted cancer therapy, and direct experimental evidence demonstrating that AI- or ML-guided design improves the performance of such platforms is currently very limited. Thus, AI/ML need to be seen at this time as a possible empowering resource for hypothesis development and design refinement instead of being an established facet of EV-oriented photomedicine. Any computationally derived predictions will require rigorous experimental and biological validation before their translational relevance can be established.

Finally, the future of EV-based photo-assisted therapy will likely be closely linked to the broader development of precision oncology. Personalized EVs generated from autologous cells, patient-specific membrane engineering, individualized cargo selection, and image-guided treatment planning may ultimately enable therapies tailored to the molecular and immunological characteristics of each patient’s tumor. Combined with advances in theranostic imaging, molecular profiling, and adaptive treatment monitoring, engineered EVs may evolve into highly personalized therapeutic systems capable of continuously integrating diagnosis, treatment, and biological feedback throughout the course of therapy.

Overall, future development of EV-based photomedicine will depend on the ability to integrate targeted delivery, biological communication, immune modulation, regulated cell death, molecular imaging, and precision oncology while maintaining reproducible manufacturing, safety, and regulatory feasibility. Progress in these areas will require coordinated advances in EV biology, nanotechnology, pharmaceutical engineering, imaging, oncology, and regulatory science. Whether increasingly engineered EV platforms can ultimately provide clinically meaningful advantages will depend on their ability to combine biological functionality with reproducible manufacturing, safety, and clearly defined therapeutic benefit.

## 8 Conclusions

EVs have emerged as a distinctive class of biologically active delivery systems for photo-assisted cancer therapy, offering capabilities that extend beyond the transport of PSs. The evidence reviewed here supports a functional evolution from EV-mediated cargo delivery toward platforms incorporating selective targeting, multimodal therapeutic integration, modulation of tumor biology and regulated cell death, and theranostic or image-guided functions. The five-generation model put forth is designed as a conceptual mechanism to arrange the escalating functional complexity, not as a definitive chronological progression or a ranking of therapeutic superiority.

At the same time, increasing engineering sophistication introduces important trade-offs in terms of heterogeneity, manufacturing reproducibility, cargo control, safety, immunogenicity, pharmacokinetics, and regulatory feasibility. Importantly, EV-based photo-assisted therapeutic platforms remain predominantly preclinical, and the field has not yet demonstrated that greater biological or engineering complexity necessarily translates into superior clinical performance. Future progress will therefore depend not only on expanding platform functionality, but also on defining which level of engineering provides the most appropriate balance between therapeutic benefit, biological robustness, manufacturability, and clinical translatability.

Taken together, EVs should be viewed not as universal replacements for synthetic nanocarriers, but as complementary biomimetic platforms whose biological properties can be selectively exploited when they provide a clear therapeutic advantage. Establishing this balance between functional innovation and translational feasibility will be essential for determining whether EV-based photo-assisted strategies can progress from experimental systems toward clinically relevant cancer therapies.

## References

[1] Foresto E Gilardi P Ibarra LE Cogno IS . Light-activated green drugs: how we can use them in photodynamic therapy and mass-produce them with biotechnological tools. Phytomed Plus. (2021) 1:100044. doi: 10.1016/j.phyplu.2021.10004442574925

[2] Al-Jamal AN Al-Hussainy AF Mohammed BA Abbas HH Kadhim IM Ward ZH . Photodynamic therapy (PDT) in drug delivery: Nano-innovations enhancing treatment outcomes. Health Sci Rev. (2025) 14:100218. doi: 10.1016/J.HSR.2025.10021842574925

[3] Cesca BA Pellicer K Martin S Caverzan MD Oliveda PM Ibarra LE . State-of-the-art photodynamic therapy for malignant gliomas: innovations in photosensitizers and combined therapeutic approaches. Explor Target Antitumor Ther. (2025) 6:1002303. doi: 10.37349/ETAT.2025.1002303 40177536 PMC11964779

[4] Ponzio RA Ibarra LE Achilli EE Odella E Chesta CA Martínez SR . Sweet light o’ mine: Photothermal and photodynamic inactivation of tenacious pathogens using conjugated polymers. J Photochem Photobiol B. (2022) 234:112510. doi: 10.1016/J.JPHOTOBIOL.2022.112510 36049287

[5] McHale AP Callan JF Nomikou N Fowley C Callan B . Sonodynamic therapy: Concept, mechanism and application to cancer treatment. Adv Exp Med Biol. (2016) 880:429–50. doi: 10.1007/978-3-319-22536-4_22 26486350

[6] Martínez SR Odella E Ibarra LE Sosa Lochedino A Wendel AB Durantini AM . Conjugated polymer nanoparticles as sonosensitizers in sono-inactivation of a broad spectrum of pathogens. Ultrasonics. (2024) 137:107180. doi: 10.1016/J.ULTRAS.2023.107180 37847942

[7] Kobayashi H Furusawa A Rosenberg A Choyke PL . Near-infrared photoimmunotherapy of cancer: a new approach that kills cancer cells and enhances anti-cancer host immunity. Int Immunol. (2021) 33:7–15. doi: 10.1093/INTIMM/DXAA037 32496557 PMC7771006

[8] Zhou H Tang D Yu Y Zhang L Wang B Karges J . Theranostic imaging and multimodal photodynamic therapy and immunotherapy using the mTOR signaling pathway. Nat Commun. (2023) 14:5350. doi: 10.1038/S41467-023-40826-5 37660174 PMC10475087

[9] Duan X Xue B Xu Z Niu Z . Multimodal photodynamic therapy by inhibiting the Nrf2/ARE signaling pathway in tumors. ACS Biomater Sci Eng. (2024) 10:7018–29. doi: 10.1021/ACSBIOMATERIALS.4C01643 39417585

[10] Millard M Yakavetsa I Piffoux M Brun A Gazeau F Guigner JM . mTHPC-loaded extracellular vesicles outperform liposomal and free mTHPC formulations by an increased stability, drug delivery efficiency and cytotoxic effect in tridimensional model of tumors. Drug Delivery. (2018) 25:1790–801. doi: 10.1080/10717544.2018.1513609 30785308 PMC6292368

[11] Ibarra LE Camorani S Agnello L Pedone E Pirone L Chesta CA . Selective photo-assisted eradication of triple-negative breast cancer cells through aptamer decoration of doped conjugated polymer nanoparticles. Pharmaceutics. (2022) 14:626. doi: 10.3390/pharmaceutics14030626 35336001 PMC8955042

[12] Krishnaswami V Loushambam B Subramanian S Raja J Vijayaraghavalu S . Nanotechnology-enhanced photosensitizers: Review of multifunctional theranostics for precision medicine. Curr Pharm Des. (2026) 32:in press. doi: 10.2174/0113816128404964251128150708 41837589

[13] Ibarra LE . Development of nanosystems for active tumor targeting in photodynamic therapy. Ther Delivery. (2022) 13:71–4. doi: 10.4155/tde-2021-0083 34842453

[14] Zhang Y Qiu Y Karimi AB Smith BR . Systematic review: Mechanisms of photoactive nanocarriers for imaging and therapy including controlled drug delivery. Eur J Nucl Med Mol Imaging. (2025) 52:1576–95. doi: 10.1007/S00259-024-07014-Z 39722062 PMC11849580

[15] Piffoux M Silva AKA Lugagne JB Hersen P Wilhelm C Gazeau F . Extracellular vesicle production loaded with nanoparticles and drugs in a trade-off between loading, yield and purity: Towards a personalized drug delivery system. Adv Biosyst. (2017) 1:1700044. doi: 10.1002/ADBI.201700044 32646153

[16] Nguyen LNM Ngo W Lin ZP Sindhwani S MacMillan P Mladjenovic SM . The mechanisms of nanoparticle delivery to solid tumours. Nat Rev Bioeng. (2024) 2:201–13. doi: 10.1038/s44222-024-00154-937880705

[17] Forgham H Chang Y Wang Y Zhu J Liu L Biggs H . The evolution of nanomedicine: the rise of next-generation nanomaterials in cancer nanomedicine. Sci Adv. (2025) 11:eadx1576. doi: 10.1126/SCIADV.ADX1576 41124269 PMC12542967

[18] Ibarra LE . Cellular trojan horses for delivery of nanomedicines to brain tumors: where do we stand and what is next? Nanomedicine. (2021) 16:517–22. doi: 10.2217/nnm-2021-0034 33634710

[19] Pagotto S Simeone P Brocco D Catitti G De Bellis D Vespa S . CAR-T-derived extracellular vesicles: A promising development of CAR-T anti-tumor therapy. Cancers 2023 Vol 15 Page 1052. (2023) 15:1052. doi: 10.3390/CANCERS15041052 36831396 PMC9954490

[20] Xie Y Mi X Xing Y Dai Z Pu Q . Past, present, and future of exosomes research in cancer: A bibliometric and visualization analysis. Hum Vaccin Immunother. (2025) 21:2488551. doi: 10.1080/21645515.2025.2488551 40207548 PMC11988232

[21] Jeppesen DK Zhang Q Franklin JL Coffey RJ . Extracellular vesicles and nanoparticles: emerging complexities. Trends Cell Biol. (2023) 33:667–81. doi: 10.1016/j.tcb.2023.01.002 36737375 PMC10363204

[22] Wu J Jin Z Fu T Qian Y Bian X Zhang X . Extracellular vesicle-based drug delivery systems in cancer therapy. Int J Mol Sci. (2025) 26:4835. doi: 10.3390/IJMS26104835 40429976 PMC12112466

[23] Fuhrmann G Serio A Mazo M Nair R Stevens MM . Active loading into extracellular vesicles significantly improves the cellular uptake and photodynamic effect of porphyrins. J Controlled Release. (2015) 205:35–44. doi: 10.1016/J.JCONREL.2014.11.029 25483424

[24] Wang B Zhang H An J Zhang Y Sun L Jin Y . Sequential intercellular delivery nanosystem for enhancing ROS-induced antitumor therapy. Nano Lett. (2019) 19:3505–18. doi: 10.1021/ACS.NANOLETT.9B00336 31034238

[25] Li R Gong X Hong C Wang H Chen Y Tan K . An efficient photochemotherapy nanoplatform based on the endogenous biosynthesis of photosensitizer in macrophage-derived extracellular vesicles. Biomaterials. (2021) 279:121234. doi: 10.1016/J.BIOMATERIALS.2021.121234 34736152

[26] Wan Z Gan X Mei R Du J Fan W Wei M . ROS triggered local delivery of stealth exosomes to tumors for enhanced chemo/photodynamic therapy. J Nanobiotechnology 2022 20:1. (2022) 20:385. doi: 10.1186/S12951-022-01591-7 35999549 PMC9400243

[27] Tao B Du R Zhang X Jia B Gao Y Zhao Y . Engineering CAR-NK cell derived exosome disguised nano-bombs for enhanced HER2 positive breast cancer brain metastasis therapy. J Controlled Release. (2023) 363:692–706. doi: 10.1016/J.JCONREL.2023.10.007 37813124

[28] Huis in ‘t Veld RV Lara P Jager MJ Koning RI Ossendorp F Cruz LJ . M1-derived extracellular vesicles enhance photodynamic therapy and promote immunological memory in preclinical models of colon cancer. J Nanobiotechnol. (2022) 20:252. doi: 10.1186/S12951-022-01448-Z 35658868 PMC9164362

[29] Zhu Z Liu Q Zhu K Wang K Lin L Chen Y . Aggregation-induced emission photosensitizer/bacteria biohybrids enhance Cerenkov radiation-induced photodynamic therapy by activating anti-tumor immunity for synergistic tumor treatment. Acta Biomater. (2023) 167:519–33. doi: 10.1016/J.ACTBIO.2023.06.009 37328041

[30] Mei J Pan W Li B Zhong M Shi X Cheng Y . Photosynthetic plant-derived nanovesicles precisely amplify photodynamic effect by light-activated oxygen generation for enhanced cancer photoimmunotherapy. ACS Nano. (2025) 19:35933–50. doi: 10.1021/ACSNANO.5C13350 41025594

[31] Yu D Ding Q Xiang C Wang D Hu L Wang J . NIR-II engineered exosome nanotheranostic probes for “oriented blasting” in orthotopic glioblastoma. ACS Nano. (2025) 19:22900–13. doi: 10.1021/ACSNANO.5C01541 40539986

[32] Zhang H Feng K Han M Shi Y Zhang Y Wu J . Homologous magnetic targeted immune vesicles for amplifying immunotherapy via ferroptosis activation augmented photodynamic therapy against glioblastoma. J Controlled Release. (2025) 383:113816. doi: 10.1016/J.JCONREL.2025.113816 40334815

[33] Ibarra LE Porcal G Macor LP Ponzio RA Spada RM Lorente C . Metallated porphyrin doped conjugated polymer nanoparticles for efficient PDT of brain and colorectal tumor cells. Nanomedicine (Lond). (2018) 13:605–24. doi: 10.2217/nnm-2017-0292 29376764

[34] Cheng X Gao J Ding Y Lu Y Wei Q Cui D . Multi-functional liposome: a powerful theranostic nano-platform enhancing photodynamic therapy. Adv Sci. (2021) 8:2100876. doi: 10.1002/ADVS.202100876 34085415 PMC8373168

[35] Zhao W Wang L Zhang M Liu Z Wu C Pan X . Photodynamic therapy for cancer: Mechanisms, photosensitizers, nanocarriers, and clinical studies. MedComm (Beijing). (2024) 5:e603. doi: 10.1002/MCO2.603 38911063 PMC11193138

[36] Gharatape A Sayadmanesh A Sadeghi-Abandansari H Ghanbari H Basiri M Faridi-Majidi R . Synthesis, characterization, and evaluation of low molecular weight poly(β-amino ester) nanocarriers for enhanced T cell transfection and gene delivery in cancer immunotherapy. Nanoscale Adv. (2025) 7:3676–91. doi: 10.1039/D5NA00169B 40342809 PMC12057721

[37] Rajagopal N Das I Kapur A Nayak S Reddy Y Sundara BK . Photodynamic therapy meets nanotechnology: A synergistic approach for cancer treatment. Transl Oncol. (2025) 62:102571. doi: 10.1016/J.TRANON.2025.102571 41109207 PMC12554145

[38] Mitchell MJ Billingsley MM Haley RM Wechsler ME Peppas NA Langer R . Engineering precision nanoparticles for drug delivery. Nat Rev Drug Discov. (2021) 20:101–24. doi: 10.1038/S41573-020-0090-8 33277608 PMC7717100

[39] Calixto GMF Bernegossi J De Freitas LM Fontana CR Chorilli M Grumezescu AM . Nanotechnology-based drug delivery systems for photodynamic therapy of cancer: a review. Molecules. (2016) 21:342. doi: 10.3390/MOLECULES21030342 26978341 PMC6274468

[40] Udrea AM Smarandache A Dinache A Mares C Nistorescu S Avram S . Photosensitizers-loaded nanocarriers for enhancement of photodynamic therapy in melanoma treatment. Pharmaceutics 2023 Vol 15. (2023) 15:15. doi: 10.3390/PHARMACEUTICS15082124 37631339 PMC10460031

[41] Gharatape A Amanzadi B Mohamadi F Rafieian M Faridi-Majidi R . Recent advances in polymeric and lipid stimuli-responsive nanocarriers for cell-based cancer immunotherapy. Nanomedicine. (2024) 19:2655–78. doi: 10.1080/17435889.2024.2416377 39540464

[42] Caverzan MD Cesca BA Palacios RE Chesta CA Ibarra LE . Transferrin-functionalized conjugated polymer nanoparticles for enhanced photodynamic therapy of glioblastoma. Int J Nanomedicine. (2026) 21:1–26. doi: 10.2147/IJN.S592688 42199657 PMC13200204

[43] Bai X Wang J Mu Q Su G . *In vivo* protein corona formation: Characterizations, effects on engineered nanoparticles’ biobehaviors, and applications. Front Bioeng Biotechnol. (2021) 9:646708. doi: 10.3389/fbioe.2021.646708 PMC8044820 33869157

[44] Hulugalla K Shofolawe-Bakare O Toragall VB Mohammad SA Mayatt R Hand K . Glycopolymeric nanoparticles enrich less immunogenic protein coronas, reduce mononuclear phagocyte clearance, and improve tumor delivery compared to PEGylated nanoparticles. ACS Nano. (2024) 18:30540–60. doi: 10.1021/ACSNANO.4C08922 39436672 PMC12045476

[45] Chen L Deng X Shen Q Chen M Li C Wang S . Novel dual strategy based on EPR/AT for optimizing therapeutic effect by improving drug delivery system physicochemical properties and regulating TME. Int J Nanomedicine. (2026) 21:1–26. doi: 10.2147/IJN.S559763 41858577 PMC12998905

[46] Lins A Fortmann M Mulac-Hahnen D Humpf HU Langer K . The nanoparticle protein corona impacts interactions with monocytes and macrophages. Int J Pharm. (2026) 690:126518. doi: 10.1016/J.IJPHARM.2025.126518 41429370

[47] Cheng H Fan JH Zhao LP Fan GL Zheng RR Qiu XZ . Chimeric peptide engineered exosomes for dual-stage light guided plasma membrane and nucleus targeted photodynamic therapy. Biomaterials. (2019) 211:14–24. doi: 10.1016/J.BIOMATERIALS.2019.05.004 31078049

[48] Russo S Giannoula Y Feola S Cerioni J Hamdan F Chiaro J . Effect of extracellular vesicles in remodeling the tumor microenvironment by DNMT1 downregulation for enhanced cancer immunotherapy. J Immunother Cancer. (2025) 13:12138. doi: 10.1136/JITC-2025-012138 41224489 PMC12612775

[49] Garofalo M Villa A Rizzi N Kuryk L Rinner B Cerullo V . Extracellular vesicles enhance the targeted delivery of immunogenic oncolytic adenovirus and paclitaxel in immunocompetent mice. J Controlled Release. (2019) 294:165–75. doi: 10.1016/J.JCONREL.2018.12.022 30557650

[50] Jo HY Kim MK Kim KT Choi C Rhee WJ . Extracellular vesicles for macrophage reprogramming: an emerging paradigm in immunomodulatory therapeutics. J Biol Eng. (2025) 20:13. doi: 10.1186/S13036-025-00594-W 41430279 PMC12837104

[51] Zhou P Du X Jia W Feng K Zhang Y . Engineered extracellular vesicles for targeted reprogramming of cancer-associated fibroblasts to potentiate therapy of pancreatic cancer. Signal Transduction Targeted Ther. (2024) 9:151. doi: 10.1038/s41392-024-01872-7 38910148 PMC11194278

[52] Sun F Ning S Fan X Wang X Lin Z Zhao J . Engineered cytomembrane nanovesicles trigger in situ storm of engineered extracellular vesicles for cascade tumor penetration and immune microenvironment remodeling. Nano Today. (2025) 61:102604. doi: 10.1016/J.NANTOD.2024.10260442574925

[53] Yang YC Jiang Q Yang KP Wang L Sethi G Ma Z . Extracellular vesicle-mediated ferroptosis, pyroptosis, and necroptosis: Potential clinical applications in cancer therapy. Cell Death Discov. (2024) 10:23. doi: 10.1038/s41420-024-01799-6 38216595 PMC10786909

[54] Aafreen S Feng J Wang W Liu G . Theranostic extracellular vesicles: a concise review of current imaging technologies and labeling strategies. Extracell Vesicles Circ Nucleic Acids. (2023) 4:107–32. doi: 10.20517/EVCNA.2023.01 37829171 PMC10568980

[55] Cesca BA Pellicer San Martin K Ibarra LE . Cellular allies against glioblastoma: therapeutic potential of macrophages and mesenchymal stromal cells. Pharmaceutics. (2026) 1:124. doi: 10.3390/pharmaceutics18010124 41599232 PMC12845474

[56] Van Niel G D’Angelo G Raposo G . Shedding light on the cell biology of extracellular vesicles. Nat Rev Mol Cell Biol. (2018) 19:213–28. doi: 10.1038/nrm.2017.125 29339798

[57] Ginini L Billan S Fridman E Gil Z . Insight into extracellular vesicle-cell communication: from cell recognition to intracellular fate. Cells. (2022) 11:1375. doi: 10.3390/CELLS11091375 35563681 PMC9101098

[58] Wang J Xing K Zhang G Li Z Ding X Leong DT . Surface components and biological interactions of extracellular vesicles. ACS Nano. (2025) 19:8433–61. doi: 10.1021/ACSNANO.4C16854 39999425

[59] Cao TGN Kang JH Kim W Lim J Kang SJ You JY . Engineered extracellular vesicle-based sonotheranostics for dual stimuli-sensitive drug release and photoacoustic imaging-guided chemo-sonodynamic cancer therapy. Theranostics. (2022) 12:1247–66. doi: 10.7150/THNO.65516 35154485 PMC8771566

[60] O’Brien K Ughetto S Mahjoum S Nair AV Breakefield XO . Uptake, functionality, and re-release of extracellular vesicle-encapsulated cargo. Cell Rep. (2022) 39:110651. doi: 10.1016/j.celrep.2022.110651 35417683 PMC9074118

[61] Bader J Brigger F Leroux JC . Extracellular vesicles versus lipid nanoparticles for the delivery of nucleic acids. Adv Drug Delivery Rev. (2024) 215:115461. doi: 10.1016/J.ADDR.2024.115461 39490384

[62] Wendler F Favicchio R Simon T Alifrangis C Stebbing J Giamas G . Extracellular vesicles swarm the cancer microenvironment: From tumor–stroma communication to drug intervention. Oncogene 2017 36:7. (2016) 36:877–84. doi: 10.1038/onc.2016.253 27546617

[63] Zhang DX Vu LT Ismail NN Le MTN Grimson A . Landscape of extracellular vesicles in the tumour microenvironment: Interactions with stromal cells and with non-cell components, and impacts on metabolic reprogramming, horizontal transfer of neoplastic traits, and the emergence of therapeutic resistance. Semin Cancer Biol. (2021) 74:24–44. doi: 10.1016/J.SEMCANCER.2021.01.007 33545339

[64] Ma D Xie A Lv J Min X Zhang X Zhou Q . Engineered extracellular vesicles enable high-efficient delivery of intracellular therapeutic proteins. Protein Cell. (2024) 15:724–43. doi: 10.1093/PROCEL/PWAE015 38518087 PMC11443452

[65] Huang Z Cheng J Deng Z Liu C Huang T Lin W . Extracellular vesicle-based therapeutic cargo delivery for cancer therapy. Int J Nanomedicine. (2025) 20:13007–37. doi: 10.2147/IJN.S548006 41179975 PMC12577471

[66] Chen C Sun M Wang J Su L Lin J Yan X . Active cargo loading into extracellular vesicles: highlights the heterogeneous encapsulation behaviour. J Extracell Vesicles. (2021) 10:e12163. doi: 10.1002/JEV2.12163 34719860 PMC8558234

[67] Kawai-Harada Y You S Scarborough T Siraj N Yedla J Rennells T . Generation of cellular biofactories for the scalable production of surface-engineered extracellular vesicles via CRISPR genome editing. ACS Biomater Sci Eng. (2026) 12(7):3821–31. doi: 10.1021/ACSBIOMATERIALS.6C00782 42340184

[68] Xu H Liu R Zhou H Kong B Shen K Zhao T . Engineered small extracellular vesicles as bioactive materials: Integrating engineering strategies for cargo loading and targeted delivery systems. Bioact Mater. (2026) 59:96–134. doi: 10.1016/J.BIOACTMAT.2025.12.029 41939725 PMC13045864

[69] Ding Y Ding H Li H Yang R Huang J Chen H . Photosensitive small extracellular vesicles regulate the immune microenvironment of triple negative breast cancer. Acta Biomater. (2023) 167:534–50. doi: 10.1016/J.ACTBIO.2023.06.004 37302734

[70] Hoseini SF Dehpour AA Bishehkolaei R Jadidi BE . Hypericin-loaded small extracellular vesicles increase photodynamic therapy and remodel miRNA–mRNA axes in AGS gastric cancer cells. Sci Rep. (2026) 16:20730. doi: 10.1038/s41598-026-48139-5 42091966 PMC13333953

[71] Lee YH Huang CY . Engineered perfluorochemical cancer-derived exosomes loaded with indocyanine green and camptothecin provide targeted photochemotherapy for effective cancer treatment. Int J Nanomedicine. (2025) 20:327–42. doi: 10.2147/IJN.S505458 39802383 PMC11725285

[72] Besbinar O Uyar R Kirbas Cilingir E Martín-Pardillos A Hueso JL Ceylan A . Cell-driven encapsulation of chlorophyllin-based carbon dots within exosomes for enhanced photodynamic therapy: miRNA profiling reveals mechanistic insights. ACS Appl Mater Interfaces. (2025) 17:66434–49. doi: 10.1021/ACSAMI.5C18555 41324588 PMC12874207

[73] Du J Wan Z Wang C Lu F Wei M Wang D . Designer exosomes for targeted and efficient ferroptosis induction in cancer via chemo-photodynamic therapy. Theranostics. (2021) 11:8185–96. doi: 10.7150/THNO.59121 34373736 PMC8344009

[74] Zhang P Chen H Liu J Liu G . Genetically engineered plasma membrane nanovesicles for cancer-targeted nanotheranostics. Methods Mol Biol. (2019) 2054:283–94. doi: 10.1007/978-1-4939-9769-5_18 31482462

[75] Yamana K Nishimura K Kawasaki R Ikeda A . Natural biomaterials in photodynamic therapy: Platforms for photosensitizer solubilization, formulation, and delivery. ChemMedChem. (2026) 21:e70312. doi: 10.1002/CMDC.70312 42174380

[76] Tian T Zhang HX He CP Fan S Zhu YL Qi C . Surface functionalized exosomes as targeted drug delivery vehicles for cerebral ischemia therapy. Biomaterials. (2018) 150:137–49. doi: 10.1016/J.BIOMATERIALS.2017.10.012 29040874

[77] Gao M Sun Q Zhang R Shan G Zhang H Peng R . Extracellular vesicles-hitchhiking boosts the deep penetration of drugs to amplify anti-tumor efficacy. Biomaterials. (2025) 314:122829. doi: 10.1016/J.BIOMATERIALS.2024.122829 39276410

[78] Lara P Huis In ‘T Veld RV Jorquera-Cordero C Chan AB Ossendorp F Cruz LJ . Zinc-phthalocyanine-loaded extracellular vesicles increase efficacy and selectivity of photodynamic therapy in co-culture and preclinical models of colon cancer. Pharmaceutics 2021 Vol 13. (2021) 13:13. doi: 10.3390/PHARMACEUTICS13101547 34683840 PMC8537141

[79] Zuo L Nie W Yu S Zhuang W Wu G Liu H . Smart tumor-cell-derived microparticles provide on-demand photosensitizer synthesis and hypoxia relief for photodynamic therapy. Angew Chem Int Ed. (2021) 60:25365–71. doi: 10.1002/ANIE.202109258 34528355

[80] Ding J Lu G Nie W Huang LL Zhang Y Fan W . Self-activatable photo-extracellular vesicle for synergistic trimodal anticancer therapy. Adv Mater. (2021) 33:2005562. doi: 10.1002/ADMA.202005562 33432702

[81] Guo R Jiang D Gai Y Qian R Zhu Z Gao Y . Chlorin e6-loaded goat milk-derived extracellular vesicles for Cerenkov luminescence-induced photodynamic therapy. Eur J Nucl Med Mol Imaging. (2022) 50:508–24. doi: 10.1007/S00259-022-05978-4 36222853

[82] Kono N Kawasaki R Oshige A Nishimura K Yamana K Yimiti D . Extracellular vesicles containing fullerene derivatives prepared by an exchange reaction for photodynamic therapy. J Mater Chem B. (2024) 12:9760–6. doi: 10.1039/D4TB00416G 39229703

[83] Liu Q Jiang D Zhang S Ru Y Li J Guo P . Light-activated photosensitizer/quercetin co-loaded extracellular vesicles for precise oral squamous cell carcinoma therapy. Int J Pharm. (2025) 671:125224. doi: 10.1016/J.IJPHARM.2025.125224 39824264

[84] Nguyen VN Nguyen Cao TG Jeong H Truong Hoang Q Pham BTT Bang J . Tumor-targeted exosome-based heavy atom-free nanosensitizers with long-lived excited states for safe and effective sono-photodynamic therapy of solid tumors. Adv Healthc Mater. (2025) 14:2500927. doi: 10.1002/ADHM.202500927 40165690 PMC12264847

[85] Feng T Tang Z Karges J Shen J Jin C Chen Y . Exosome camouflaged coordination-assembled iridium(III) photosensitizers for apoptosis-autophagy-ferroptosis induced combination therapy against melanoma. Biomaterials. (2023) 301:122212. doi: 10.1016/J.BIOMATERIALS.2023.122212 37385136

[86] Qian Y Li C Gao J Wang S Wu X Wei L . Autologous exosomes loaded with a rationally designed photosensitizer for enhanced photodynamic therapy. Chem Commun. (2024) 60:10180–3. doi: 10.1039/D4CC02168A 39190479

[87] Xu S Xie X He P Zhu S Li X Chen Q . Nitric oxide-producing multiple functional nanoparticle remodeling tumor microenvironment for synergistic photodynamic immunotherapy against hypoxic tumor. ACS Nano. (2025) 19:6371–87. doi: 10.1021/ACSNANO.4C16329 39913864

[88] Zhou H Zhu C Li Y Zhao F Feng Q Liu S . Exosome/liposome hybrid nanovesicles for enhanced phototherapy and boosted anti-tumor immunity against melanoma. Eur J Med Chem. (2025) 289:117485. doi: 10.1016/J.EJMECH.2025.117485 40081104

[89] Slivinschi B Manai F Martinelli C Carriero F D’Amato C Massarotti M . Enhanced delivery of Rose Bengal by amino acids starvation and exosomes inhibition in human astrocytoma cells to potentiate anticancer photodynamic therapy effects. Cells 2022 Vol 11. (2022) 11:11. doi: 10.3390/CELLS11162502 36010578 PMC9406355

[90] Calzoni E Bertoldi A Cesaretti A Alabed HBR Cerrotti G Pellegrino RM . Aloe extracellular vesicles as carriers of photoinducible metabolites exhibiting cellular phototoxicity. Cells. (2024) 13:1845. doi: 10.3390/CELLS13221845 39594594 PMC11592872

[91] Tu Y Dong Y Wang K Shen S Yuan Y Wang J . Intercellular delivery of bioorthogonal chemical receptors for enhanced tumor targeting and penetration. Biomaterials. (2020) 259:120298. doi: 10.1016/J.BIOMATERIALS.2020.120298 32827798

[92] Ma X Zhu X Zhu Z Ma Q Jia S Wu N . A biomimetic nanozyme engineered via coordination and confinement for photothermal-reinforced catalytic detection of extracellular vesicles and diagnosis of cancer. Anal Chem. (2026) 98:14447–59. doi: 10.1021/ACS.ANALCHEM.6C01091 42083729

[93] Yakavets I Francois A Lamy L Piffoux M Gazeau F Wilhelm C . Effect of stroma on the behavior of temoporfin-loaded lipid nanovesicles inside the stroma-rich head and neck carcinoma spheroids. J Nanobiotechnol. (2021) 19:3. doi: 10.1186/S12951-020-00743-X 33407564 PMC7789590

[94] Ma J Wen C Chen M Zhang W Wang L Yin H . Ce6/PTX2-NP/G@NHs confer radiosensitivity in non-small cell lung cancer via promotion of apoptotic body-mediated neighboring effects. ACS Biomater Sci Eng. (2023) 9:2793–805. doi: 10.1021/ACSBIOMATERIALS.2C01549 37066871

[95] Wang X Ding H Li Z Peng Y Tan H Wang C . Exploration and functionalization of M1-macrophage extracellular vesicles for effective accumulation in glioblastoma and strong synergistic therapeutic effects. Signal Transduction Targeted Ther 2022 7:1. (2022) 7:74. doi: 10.1038/s41392-022-00894-3 35292619 PMC8924195

[96] Xie L Ma F Qiu C Tong S Gong J Xie X . Nanoassemblies remodeling tumor-derived exosomes’ immunity ameliorate local and peripheral immunosuppression for cancer immunotherapy. ACS Nano. (2026) 20:17442–62. doi: 10.1021/ACSNANO.6C03357 42249854

[97] Kim S Kang JH Nguyen Cao TG Kang SJ Jeong K Kang HC . Extracellular vesicles with high dual drug loading for safe and efficient combination chemo-phototherapy. Biomater Sci. (2022) 10:2817–30. doi: 10.1039/D1BM02005F 35384946

[98] Qian R Jing B Jiang D Gai Y Zhu Z Huang X . Multi-antitumor therapy and synchronous imaging monitoring based on exosome. Eur J Nucl Med Mol Imaging 2022 49:8. (2022) 49:2668–81. doi: 10.1007/S00259-022-05696-X 35091755

[99] Nguyen Cao TG Kang JH You JY Kang HC Rhee WJ Ko YT . Safe and targeted sonodynamic cancer therapy using biocompatible exosome-based nanosonosensitizers. ACS Appl Mater Interfaces. (2021) 13:25575–88. doi: 10.1021/ACSAMI.0C22883 34033477

[100] Li Y Chen L Chen Y Shi H Yu S Funmilayo A . Exosome-decorated bio-heterojunctions reduce heat and ROS transfer distance for boosted antibacterial and tumor therapy. Biomaterials. (2025) 315:122921. doi: 10.1016/J.BIOMATERIALS.2024.122921 39467398

[101] Chen D Guo Z Yao L Sun Y Dian Y Zhao D . Targeting oxidative stress-mediated regulated cell death as a vulnerability in cancer. Redox Biol. (2025) 84:103686. doi: 10.1016/J.REDOX.2025.103686 40424719 PMC12159232

[102] Caverzan MD Ibarra LE . Advancing glioblastoma treatment through iron metabolism: a focus on TfR1 and ferroptosis innovations. Int J Biol Macromol. (2024) 278:134777. doi: 10.1016/J.IJBIOMAC.2024.134777 39153669

[103] Yang X Xie X Liu S Ma W Zheng Z Wei H . Engineered exosomes as theranostic platforms for cancer treatment. ACS Biomater Sci Eng. (2023) 9:5479–503. doi: 10.1021/ACSBIOMATERIALS.3C00745 37695590

[104] Srivastava A Rathore S Munshi A Ramesh R . Organically derived exosomes as carriers of anticancer drugs and imaging agents for cancer treatment. Semin Cancer Biol. (2022) 86:80–100. doi: 10.1016/J.SEMCANCER.2022.02.020 35192929 PMC9388703

[105] Almeida S Santos L Falcão A Gomes C Abrunhosa A . *In vivo* tracking of extracellular vesicles by nuclear imaging: Advances in radiolabeling strategies. Int J Mol Sci. (2020) 21:1–13. doi: 10.3390/IJMS21249443 33322484 PMC7764519

[106] Ou Z He X Li Q Cao N Gao M He B . High-performance tracking of bacterial extracellular vesicles in living systems using an aggregation-induced emission luminogen. Chem Eng J. (2022) 446:136847. doi: 10.1016/J.CEJ.2022.13684742574925

[107] Li Y Fan X Li Y Zhu L Chen R Zhang Y . Biologically excretable AIE nanoparticles wear tumor cell-derived “exosome caps” for efficient NIR-II fluorescence imaging-guided photothermal therapy. Nano Today. (2021) 41:101333. doi: 10.1016/J.NANTOD.2021.10133342574925

[108] Jang Y Kim H Yoon S Lee H Hwang J Jung J . Exosome-based photoacoustic imaging guided photodynamic and immunotherapy for the treatment of pancreatic cancer. J Controlled Release. (2021) 330:293–304. doi: 10.1016/J.JCONREL.2020.12.039 33359580

[109] Van Delen M Derdelinckx J Wouters K Nelissen I Cools N . A systematic review and meta-analysis of clinical trials assessing safety and efficacy of human extracellular vesicle-based therapy. J Extracell Vesicles. (2024) 13:e12458. doi: 10.1002/JEV2.12458 38958077 PMC11220457

[110] Tran HL Zheng W Issadore DA Im H Cho YK Zhang Y . Extracellular vesicles for clinical diagnostics: From bulk measurements to single-vesicle analysis. ACS Nano. (2025) 19:28021–109. doi: 10.1021/ACSNANO.5C00706 40720603 PMC12356129

[111] Nikoloff JM Saucedo-Espinosa MA Kling A Dittrich PS . Identifying extracellular vesicle populations from single cells. Proc Natl Acad Sci USA. (2021) 118:e2106630118. doi: 10.1073/PNAS.2106630118 34518226 PMC8463870

[112] Almeria C Kreß S Weber V Egger D Kasper C . Heterogeneity of mesenchymal stem cell-derived extracellular vesicles is highly impacted by the tissue/cell source and culture conditions. Cell Bioscience. (2022) 12:51. doi: 10.1186/S13578-022-00786-7 35501833 PMC9063275

[113] Welsh JA Goberdhan DCI O’Driscoll L Buzas EI Blenkiron C Bussolati B . Minimal information for studies of extracellular vesicles (MISEV2023): From basic to advanced approaches. J Extracell Vesicles. (2024) 13:e12404. doi: 10.1002/JEV2.12404 38326288 PMC10850029

[114] Preußer C Salander DJ Szymanski W Graumann J Wang Y Zhao K . Isolation defines identity: Functional consequences of extracellular vesicle purification strategies. Adv Healthc Mater. (2026) 15:e04684. doi: 10.1002/ADHM.202504684 41555698 PMC13058776

[115] Yuan R Zhou Y Arias GF Dittmer DP . Extracellular vesicle isolation by a tangential-flow filtration-based large-scale purification method. Methods Mol Biol. (2023) 2668:45–55. doi: 10.1007/978-1-0716-3203-1_5 37140789

[116] Kim YW Jeon SY Lee KM Shin S . Advanced extracellular vesicle isolation: a hybrid electrokinetic-tangential flow filtration approach for improved yield, purity, and scalability. Anal Chem. (2025) 97:16759–68. doi: 10.1021/ACS.ANALCHEM.5C01168 40736764 PMC12355454

[117] Wiest EF Zubair AC . Generation of current good manufacturing practices-grade mesenchymal stromal cell-derived extracellular vesicles using automated bioreactors. Biology. (2025) 14:14. doi: 10.3390/BIOLOGY14030313 40136569 PMC11940689

[118] Zhu X Badawi M Pomeroy S Sutaria DS Xie Z Baek A . Comprehensive toxicity and immunogenicity studies reveal minimal effects in mice following sustained dosing of extracellular vesicles derived from HEK293T cells. J Extracell Vesicles. (2017) 6:1324730. doi: 10.1080/20013078.2017.1324730 28717420 PMC5505007

[119] Xia Y Zhang J Liu G Wolfram J . Immunogenicity of extracellular vesicles. Adv Mater. (2024) 36:2403199. doi: 10.1002/ADMA.202403199 38932653

[120] Driedonks T Jiang L Carlson B Han Z Liu G Queen SE . Pharmacokinetics and biodistribution of extracellular vesicles administered intravenously and intranasally to Macaca nemestrina. J Extracell Biol. (2022) 1:e59. doi: 10.1002/JEX2.59 36591537 PMC9799283

[121] Patel S Schmidt KF Farhoud M Zi T Jang SC Dooley K . *In vivo* tracking of [89Zr]Zr-labeled engineered extracellular vesicles by PET reveals organ-specific biodistribution based upon the route of administration. Nucl Med Biol. (2022) 112–113:20–30. doi: 10.1016/J.NUCMEDBIO.2022.06.004 35763877

[122] Verma N Arora S . Navigating the global regulatory landscape for exosome-based therapeutics: Challenges, strategies, and future directions. Pharmaceutics 2025 Vol 17. (2025) 17:17. doi: 10.3390/PHARMACEUTICS17080990 40871013 PMC12389065

[123] Greenberg ZF Graim KS He M . Towards artificial intelligence-enabled extracellular vesicle precision drug delivery. Adv Drug Delivery Rev. (2023) 199:114974. doi: 10.1016/J.ADDR.2023.114974 37356623

